# Increased BUB1B/BUBR1 expression contributes to aberrant DNA repair activity leading to resistance to DNA-damaging agents

**DOI:** 10.1038/s41388-021-02021-y

**Published:** 2021-09-20

**Authors:** Kazumasa Komura, Teruo Inamoto, Takuya Tsujino, Yusuke Matsui, Tsuyoshi Konuma, Kazuki Nishimura, Taizo Uchimoto, Takeshi Tsutsumi, Tomohisa Matsunaga, Ryoichi Maenosono, Yuki Yoshikawa, Kohei Taniguchi, Tomohito Tanaka, Hirofumi Uehara, Koichi Hirata, Hajime Hirano, Hayahito Nomi, Yoshinobu Hirose, Fumihito Ono, Haruhito Azuma

**Affiliations:** 1 Department of Urology, Osaka Medical and Pharmaceutical University, Osaka, 569-8686 Japan; 2 Translational Research Program, Osaka Medical and Pharmaceutical University, Osaka, 569-8686 Japan; 3 Division of Urology, Department of Surgery, Brigham and Women’s Hospital, Harvard Medical School, Boston, MA 02115 United States; 4 Biomedical and Health Informatics Unit, Department of Integrated Health Science, Nagoya University Graduate School of Medicine, Nagoya, 461-8673 Japan; 5 Institute for Glyco-core Research (iGCORE), Nagoya University, Nagoya, 461-8673 Japan; 6 Graduate School of Medical Life Science, Yokohama City University, Yokohama, 230-0045 Japan; 7 Department of Pathology, Osaka Medical and Pharmaceutical University, Osaka, 569-8686 Japan; 8 Department of Physiology, Osaka Medical and Pharmaceutical University, Osaka, 569-8686 Japan

**Keywords:** Cancer therapeutic resistance, Bladder cancer, DNA damage response

## Abstract

There has been accumulating evidence for the clinical benefit of chemoradiation therapy (CRT), whereas mechanisms in CRT-recurrent clones derived from the primary tumor are still elusive. Herein, we identified an aberrant BUB1B/BUBR1 expression in CRT-recurrent clones in bladder cancer (BC) by comprehensive proteomic analysis. CRT-recurrent BC cells exhibited a cell-cycle-independent upregulation of BUB1B/BUBR1 expression rendering an enhanced DNA repair activity in response to DNA double-strand breaks (DSBs). With DNA repair analyses employing the CRISPR/cas9 system, we revealed that cells with aberrant BUB1B/BUBR1 expression dominantly exploit mutagenic nonhomologous end joining (NHEJ). We further found that phosphorylated ATM interacts with BUB1B/BUBR1 after ionizing radiation (IR) treatment, and the resistance to DSBs by increased BUB1B/BUBR1 depends on the functional ATM. In vivo, tumor growth of CRT-resistant T24R cells was abrogated by ATM inhibition using AZD0156. A dataset analysis identified FOXM1 as a putative BUB1B/BUBR1-targeting transcription factor causing its increased expression. These data collectively suggest a redundant role of BUB1B/BUBR1 underlying mutagenic NHEJ in an ATM-dependent manner, aside from the canonical activity of BUB1B/BUBR1 on the G2/M checkpoint, and offer novel clues to overcome CRT resistance.

## Introduction

Emerging evidence for the administration of chemoradiation therapy (CRT) with curative intent has been increasingly reported in the treatment of various types of cancers [1]. DNA double-strand breaks (DSBs) induced by ionizing radiation (IR) or genotoxic agents represent a highly critical form of DNA damage, which must be repaired to maintain genomic integrity and is closely associated with tumorigenesis. Cells inherently use two general types of systems to repair DSBs. The first set of the mechanism is referred to as nonhomologous end joining (NHEJ) thought to be a predominant repair pathway for DSBs. NHEJ is characterized by the direct ligation of broken ends at the site of DSBs without the use of an extensive homologous template. The other repair pathway is termed homologous recombination (HR), which is mainly mediated in S and G2 phases when the identical sister chromatids exist as a template. Each repair pathway is activated through the interaction of a distinct set of proteins, thereby frequently leading to different consequences at the site of DSBs such as indels [2]. In particular, it is now clear that NHEJ is classified into at least two models, referred to as classical nonhomologous end joining (C-NHEJ) and alternative nonhomologous end joining (A-NHEJ). It has been well-documented that C-NHEJ utilizes the core factors, including KU (KU70/80), DNA-PKcs, and DNA ligase IV (LIG4) [3], and recent studies have indicated that the consequence of C-NHEJ at DSBs is mostly error-free [4, 5]. On the other hand, A-NHEJ has been considered error-prone and mutagenic with various factors contributing to this repair mechanism, although the molecular basis of this is still poorly understood.

Herein, we investigated pair-matched clinical BC samples (treatment-naïve and CRT-recurrent tumors) from the same patient and identified an aberrant BUB1B/BUBR1 expression level in CRT-recurrent clone, which facilitates mutagenic NHEJ activity in response to IR and cisplatin leading to the tumor harboring accumulated mutations.

## Materials and methods

All the experiments were performed with the approval of the institutional review board (IRB), i.e., approved No. RIN-25(2305) (Takatsuki, Osaka, Japan). Animal experiments were conducted with approval from the Institutional Animal Care and Use Committee of Osaka Medical and Pharmaceutical University: approval No. 29101 N (Takatsuki, Osaka, Japan). All the additional information can be found in “Supplementary Methods” [6–9]. All the materials used in the present study are listed in “Supplementary Materials”.

## Results

### Overexpression of BUB1B/BUBR1 in chemo-radiation resistant human bladder cancer

**Fig. 1:**
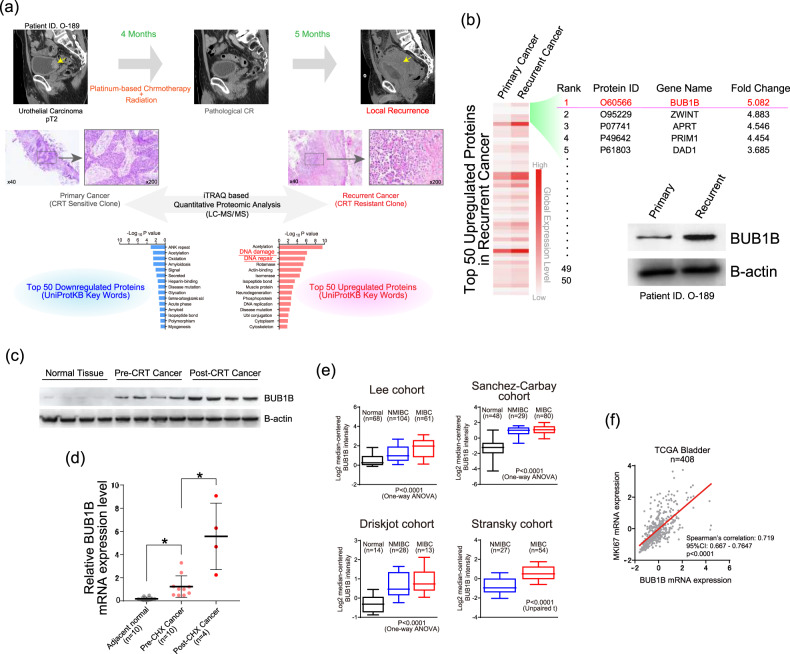
Overexpression of BUB1B/BUBR1 in chemo-radiation therapy (CRT)-resistant human bladder cancer. **a** Comprehensive proteomic analysis by mass spectrometry (MS) with tandem mass tag (TMT)-labelling quantification was performed using pair-matched clinical samples from the same bladder cancer (BC) patient (primary tumor and CRT-resistant tumor). Top 50 downregulated and up-regulated proteins were applied to gene-ontology (GO) term analysis for UniProtKB keywords. Note that DNA damage and DNA repair pathways were significantly up-regulated in CRT-resistant tumors. **b** Heatmap showing the difference of top 50 upregulated proteins in CRT-recurrent clone. BUB1B/BUBR1 was identified as the top candidate of up-regulated protein, and immunoblotting of the corresponding clinical samples confirmed the upregulation in CRT-recurrent tumor. **c** Immunoblotting of the clinical samples in BC patients, including normal bladder tissue, pre-CRT tumor, and post-CRT tumor. B-actin was loaded as an internal control. **d** qPCR of BUB1B/BUBR1 expression levels among indicated clinical samples, including normal bladder tissue, pre-CRT tumor, and post-CRT tumor. Ct values were normalized by GAPDH. Data are expressed as relative mean-fold change (mean ± SD). * indicates *p* < 0.05. **e** BUB1B/BUBR1 mRNA expression level in normal bladder tissue, non-muscle-invasive BC (NMIBC), and muscle-invasive BC (MIBC) among four publicly available datasets [27, 34, 46, 47]. **f** The correlation between BUB1B/BUBR1 and MKI67 mRNA expression level in The Cancer Genome Atlas (TCGA) dataset [10]. Linear regression analysis was performed to examine Spearman’s correlation coefficient.

To explore the mechanism by which BC cells acquire the resistance to CRT, we first performed the comprehensive proteomic analysis by tandem mass tag (TMT)-labelling quantification of mass spectrometry in clinical patient samples (Fig. 1a). Pair-matched samples from the same patients (pre-CRT primary tumor and CRT-recurrent tumor) were analyzed. In total, 1040 proteins were identified in both samples. We then extracted the top 50 upregulated and downregulated proteins in CRT-recurrent tumor compared with the primary treatment-naive tumor (Supplementary Tables 1, 2). Pathway analysis by gene ontology (GO) in those proteins showed that DNA repair-related pathways in UniProtKB Key Words were listed for the top 50 upregulated proteins in CRT-recurrent tumor. Of note, we found BUB1B/BUBR1 as the most upregulated protein in CRT-recurrent tumor as compared with the primary treatment-naïve tumor (Fig. 1b) and immunoblotting from the corresponding samples confirmed the upregulation of BUB1B/BUBR1 in CRT-recurrent tumor. Next, we examined an expression level of BUB1B/BUBR1 using other BC patient samples. Increased BUB1B/BUBR1 protein-expression level in CRT-recurrent tumor seemed to be evident compared with the normal bladder and primary tumor tissue (Fig. 1c), and mRNA expression level was also significantly upregulated in the CRT-recurrent tumor (Fig. 1d). These data indicated that increased BUB1B/BUBR1 protein expression in the CRT-recurrent BC cells is at least in part due to its increased mRNA expression level.

We also investigated the mRNA expression level of BUB1B/BUBR1 in publicly available datasets. Increased BUB1B/BUBR1 mRNA expression level with disease progression, namely the highest expression in muscle-invasive BC (MIBC) compared with non-muscle-invasive BC (NMIBC), was confirmed (Fig. 1e). BUB1B/BUBR1 mRNA expression level was positively correlated with MKI67 expression level in the TCGA BC dataset [10], known as a proliferation marker (Fig. 1f). These data collectively suggested an aggressive property with increased BUB1B/BUBR1 expression level in BC patients.

### Resistance to IR and cisplatin by increased BUB1B/BUBR1 expression in T24R and JMSU1R BC cells

**Fig. 2:**
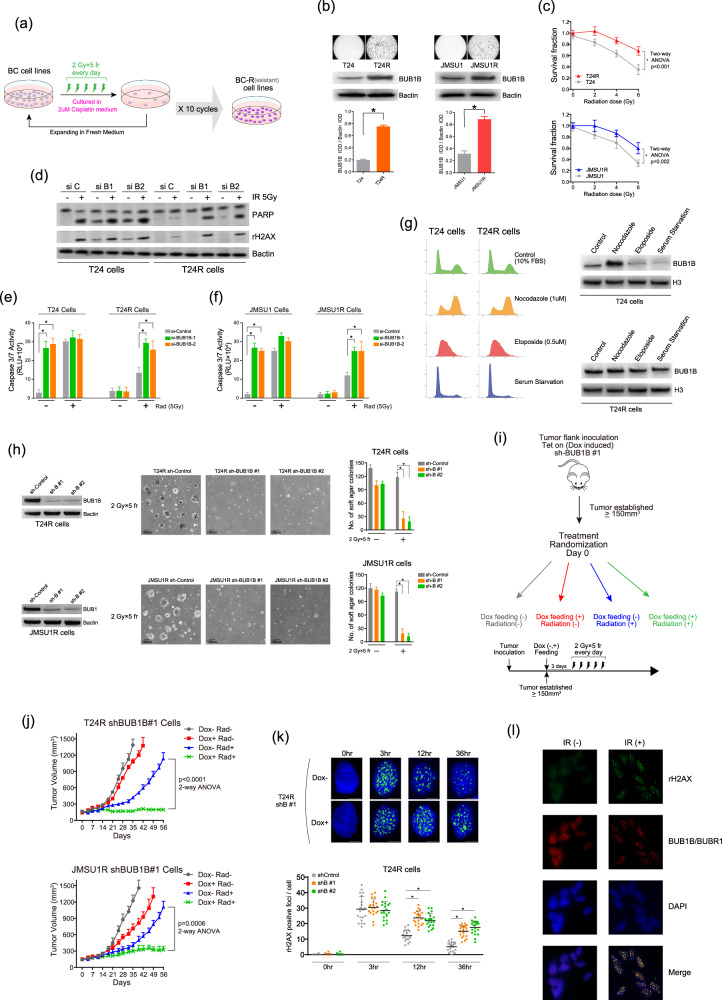
Resistance to IR and cisplatin by increased BUB1B/BUBR1 expression in T24R and JMSU1R BC cells. **a** Schematic representation of the protocol for the establishment of T24R and JMSU1R CRT-resistant BC cell lines. Parent T24 and JMSU1 BC cell lines were treated with IR (2 Gy/5 fraction × 10 cycle: total 50 Gy) and 2 µM of cisplatin. **b** Soft-agar colony-formation assay in T24, T24R, JMSU1, and JMSU1R cell lines treated with 1 µM of cisplatin. The medium was changed every 3days, and representative images after 21 days are shown. Lower panels exhibit immunoblotting in indicated cell lines. B-actin was loaded as an internal control. Quantitative evaluation by integrated optical density (IOD) for the immunoblotting was performed in three independent experiments, and the results are shown as mean + SD. *P < 0.05, unpaired *t*-test. **c** BC cell lines were treated with IR in the indicated dose, followed by the measurement of cell-viability assay after six days. The inhibitory effect on cell growth by the IR is presented as a relative value (mean ± SD) compared with control (0 Gy) as 100%. **d** Immunoblotting in indicated cell lines transfected with siRNAs three days before the IR treatment. Cells were collected 24 h after the IR treatment. B-actin was loaded as an internal control. **e**, **f** Cells were transfected with indicated siRNAs and incubated for three days. Thereafter, those cells were treated with or without 5 Gy of IR. Two days later, caspase 3/7 activity was measured. * indicates *p* < 0.05. **g** Left panel: cell-cycle analysis in T24 and T24R cells. Cell-cycle synchronization was performed by serum starvation in 0.1% FBS (G1 phase), 0.5 µM of etoposide (S phase), and 1 µM of nocodazole (G2/M phase) for 36 h. Right panel: Immunoblotting in T24 and T24R cell lines. Nuclear fractions incubated with indicated drugs for cell-cycle synchronization were subjected to immunoblotting with the indicated antibodies. Histone 3 was loaded as an internal control. **h** Immunoblotting of shControl and shBUB1B#1,2 in T24R and JMSU1R cell lines. Cells were cultured with 0.15 μg/ml of doxycycline for three days, then subjected to immunoblotting with indicated antibodies—right panel: Soft-agar colony-formation assay in these cells. Cells were cultured with 0.15 μg/ml of doxycycline for three days, then treated with IR (2 Gy/5fr every day). The medium was changed every 3days. Representative images are shown after 21 days. The number of colonies was counted in five random fields in 21 days, and the results are shown as mean ± SD. * indicates *p* < 0.05. **i** Schematic of the protocol for the xenograft mouse model. After tumors developed reaching 150 mm^3^ of tumor volume, mice were randomized into four groups with five mice in each group. **j** Tumor growth of T24R shBUB1B#1 and JMSU1R shBUB1B#1 cells in the xenograft mouse model treated with or without IR and doxycycline feeding. The result is shown as mean ± SD. **k** Representative images of γ-H2AX-positive foci induced by 5 Gy of IR in T24R sh-BUB1B#1 cells with or without 0.15 μg/ml of doxycycline. Scale bar indicates 10 μm. The bottom panels show the quantification of the number of γ-H2AX-positive foci among indicated cells cultured with 0.15 µg/ml of doxycycline. The results are shown as mean ± SD. * indicates *p* < 0.05. **l** Immunofluorescence of double staining with rH2AX and BUB1B/BUBR1 antibodies in T24R cells. Cells were treated with or without 6 Gy IR treatment, then fixed three hours after the treatment.

We sought to develop CRT-resistant clones in BC cell lines. T24 and JMSU1 BC cell lines were treated with IR (2 Gy/5 fraction × 10 cycle: total 50 Gy) and 2 µM of cisplatin, and CRT-resistant clone was established as T24R and JMSU1R cell lines (Fig. 2a). These CRT-resistant cell lines showed decreased sensitivity to cisplatin and IR with increased BUB1B/BUBR1 protein expression level compared with the parent cells (Fig. 2b, c). Overexpression of BUB1B/BUBR1 to the parent T24 and JMSU1 cells resulted in the decreased sensitivity to the IR treatment (Supplementary Figure 1a, b). In contrast, knockdown of BUB1B/BUBR1 in the parent cells (T24 and JMSU1 cells) exhibited an increased cleaved PARP expression and caspase3/7 activity compared with the si-Control implying massive apoptosis by the knockdown of BUB1B/BUBR1, whereas there seemed to be no difference of cleaved PARP expression and caspase3/7 activity between si-Control and si-BUB1B in the resistant T24R and JMSU1R cells (Fig. 2d–f, Supplementary Figure 1c, d). We also found that the knockdown of BUB1B/BUBR1 reversed the resistance to cisplatin in these T24R and JMSU1R cells (Supplementary Figure 1e).

BUB1B/BUBR1 has been reported as a component of mitotic-checkpoint complex (MCC) and an inhibitor of anaphase-promoting complex/cyclosome (APC/C) [11–13]. Therefore, we assessed whether the knockdown of BUB1B/BUBR1 changes the cell growth and cell cycle. Interestingly, knockdown of BUB1B/BUBR1 inhibited cell growth and altered cell cycle in JMSU1R cells, but not in T24R cells (Supplementary Figure 1f, g). Since previous studies have reported that BUB1B/BUBR1 expression level is tightly regulated in a cell cycle-dependent manner [14, 15], we performed cell synchronization in T24R and parent T24 cells, and examined BUB1B/BUBR1 expression level in particular cell-cycle phase (Fig. 2g). Of note, BUB1B/BUBR1 protein-expression level was specifically up-regulated in the G2/M phase in parent T24 cells, whereas T24R cells constitutively exhibited increased BUB1B/BUBR1 expression level across the cell-cycle phases.

To further explore the phenotype, Tet-on-induced sh-BUB1B/BUBR1 system was utilized using lentivirus in T24R and JMSU1R cells (Fig. 2h). Soft-agar colony-formation assay exhibited that T24R and JMSU1R sh-Control cells showed anchorage-independent growth after 2 Gy x 5fr of IR, while those cells with sh-BUB1#1,2 showed a limited growth under the same treatment. Next, we adopted the xenograft mouse model. The knockdown of BUB1B/BUBR1 was induced by feeding 0.1% doxycycline three days before the initiation of the IR (2 Gy x 5fr: total 10 Gy) (Fig. 2i). Without knockdown of BUB1B/BUBR1, T24R and JMSU1R cells developed tumors against the IR treatment, whereas the IR-resistant growth was abrogated by the knockdown of BUB1B/BUBR1 in both xenograft models (Fig. 2j).

We next compared the number of rH2AX-positive foci representing the extent of DSBs following the IR in these cells. There seemed to be comparable positive foci three hours after the IR between sh-control and sh-BUB1B/BUBR1, whereas knockdown of BUB1B/BUBR1 exhibited a significantly higher level of rH2AX in 12 and 36 h after the IR compared with sh-control (Fig. 2k, Supplementary Figure 1h). In addition, we performed immunofluorescence of double staining with rH2AX and BUB1B/BUBR1 antibodies in the resistant T24R cells that express an increased BUB1B/BUBR1 expression level. Importantly, rH2AX and BUB1B/BUBR1-positive foci were colocalized with the IR treatment (Fig. 2l). These data indicated that an aberrant BUB1B/BUBR1 expression renders an enhanced DNA repair activity in response to DSBs, as proven by the sustained rH2AX positive foci in the knockdown of BUB1B/BUBR1 expression level and the colocalization of rH2AX- and BUB1B/BUBR1 in response to DSBs.

### Increased BUB1B/BUBR1 expression promotes mutagenic NHEJ

**Fig. 3:**
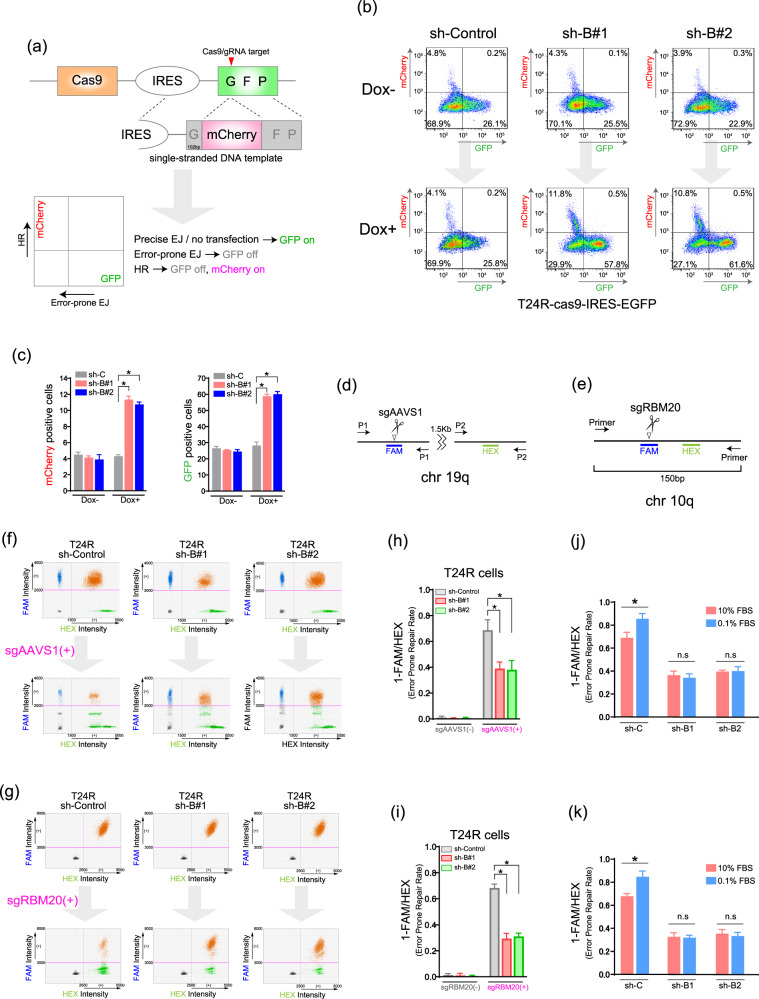
Increased BUB1B/BUBR1 expression promotes mutagenic NHEJ. **a** The reporter assay protocol to quantify nonhomologous endjoining (NHEJ) and homologous recombination (HR) using the CRISPR/cas9 system. Lentiviral infection (TRE–KRAB–dCas9–IRES–GFP) was performed to stably express dCas9 and integrate DNA sequence (IRES–GFP). The vector of sgRNA-targeting GFP with U6 promotor and synthesized single-stranded DNA (ssDNA) for HR repair (knock-in donor) were simultaneously transfected to cells stably expressing dCas9. **b** Flow cytometry of GFP- and mCherry-positive cells. T24R–cas9–IRES–EGFP shControl and shBUB1B#1, 2 cells were cultured with or without 0.15 µg/ml of doxycycline for 3days, then transfected with sgRNA targeting EGFP and single-stranded DNA template. Cells were collected and analyzed using flow cytometry three days after the transfection. **c** The proportion of GFP- and mCherry-positive cells in T24R–cas9–IRES–EGFP shControl and shBUB1B#1, 2 cells. The results are shown as mean + SD. *P < 0.05, unpaired *t*-test. **d**, **e** Schematic representation of the reporter assay using digital-droplet PCR (ddPCR). Cells were transfected with CRISPR ribonucleoprotein (RNP) complex targeting genomic region and incubated for 72 h. Probes were designed on sgRNA-targeted sites for FAM reporters and reference sites for HEX reporters. **f**, **g** The result of ddPCR reporter assay in T24R shControl and shBIB1B#1, 2 cells. Cells were cultured with 0.15 µg/ml of doxycycline for 72 h, followed by the transfection of the CRISPR ribonucleoprotein (RNP) complex with or without sgRNA. Three days after the transfection, cells were harvested and analyzed. **h**, **i** The rate of mutagenic repair after the sgRNA transfection (defined as 1-FAM/HEX). The results are shown as mean + SD. *P < 0.05, unpaired *t*-test. **j**, **k** The rate of mutagenic repair after the sgRNA transfection (defined as 1-FAM/HEX) in T24R-sh C (control), B1 (BUB1B#1), and B2 (BUB1B#2). For the serum starvation, cells were cultured with 0.15 µg/ml of doxycycline (10% FBS) for 24 h, then the medium was changed to 0.1% FBS, including 0.15 µg/ml of doxycycline for 48 h. Thereafter, the transfection of CRISPR ribonucleoprotein (RNP) complex with or without sgRNA was performed in a 10% FBS medium. Results are shown as mean + SD. *P < 0.05, unpaired *t*-test.

Since the data suggested that increased BUB1B/BUBR1 expression affects the repair of DSBs in BC cells, we next sought to uncover the biological mechanism. First, we developed a methodology to quantify nonhomologous endjoining (NHEJ) and homologous recombination (HR) using the CRISPR/cas9 system. As shown in Fig. 3a, lentiviral infection of TRE–KRAB–dCas9–IRES–GFP was performed to stably express dCas9 and integrate DNA sequence (IRES-GFP). The vector of sgRNA-targeting GFP with U6 promotor and synthesized single-stranded DNA (ssDNA) for HR repair (knock-in donor) was simultaneously transfected to cells stably expressing dCas9. We first used 293 T cells with or without BUB1B/BUBR1 overexpression in the analysis (Supplementary Figure 2a). Flow cytometry of GFP- and mCherry- positive cells were analyzed in which decreased GFP and increased mCherry-positive cells denote mutagenic NHEJ and HR, respectively (Supplementary Figure 2b). Interestingly, this experiment revealed that overexpression of BUB1B/BUBR1 in 293 T cells resulted in a decreased ratio of both mCherry- and GFP-positive cells as compared with the parent 293 T cells (Supplementary Figure 2c). We next examined whether knockdown of endogenous BUB1B/BUBR1 in T24R cells varies the ratio of GFP- and mCherry-positive cells (Fig. 3b). As shown in Fig. 2c, without knockdown of BUB1B/BUBR1, the ratio of GFP and mCherry positive cells after the transfection of sgRNA and ssDNA was similar among sh-control, BUB1B/BUBR1#1, 2, which indicates that transfection efficiency was comparable between these shRNAs in T24R cells. Of note, an addition of 0.15 μg/ml doxycycline inducing shRNA transcription exhibited an increased ratio of both GFP- positive and mCherry-positive cells in shBUB1B/BUBR1#1 and #2 compared with shControl. These data suggest that consistent with the result of overexpression of BUB1B/BUBR1 in 293 T cells (Supplementary Figure 2b, c), cells with aberrant BUB1B/BUBR1 expression dominantly exploit mutagenic NHEJ rather than precise NHEJ or HR in response to DSBs.

We next explored the DNA repair process in endogenous genomic sites in T24R and JMSU1R cells. As shown in Fig. 3d, e, digital-droplet PCR was employed to determine the absolute number of indels by mutagenic NHEJ (FAM probe) comparing the internal-control site (HEX probe) on AAVS1(Chr 19) and RBM20 (chr10) in response to the sgRNA transfection [16, 17]. In short, we designed a FAM probe on CRISPR/cas9-cleavage site, and the HEX probe is used for the baseline control. Theoretically, without the sgRNA transfection, there are supposed to be the same copy numbers between adjacent two genomic loci recognized by FAM and HEX probes (FAM / HEX = 1). If the cleavage efficiency were perfect, FAM/HEX ratio would depict the ratio of accurate DNA repair, e.g., “FAM/HEX = 1” in the case of all accurate DNA repair. Although the cleavage efficiency and the ratio of accurate DNA repair were not identical, the focus in this experiment was the count of FAM probe after sgRNA transfection that can offer the absolute number of mutagenic DNA repair with the comparison of the control HEX probe. Thus, decreased FAM count represents the event of indels, which offers the proportion of DNA repair by mutagenic NHEJ calculated as (1-FAM/HEX) (Figs. 3f, g, Supplementary Figure 2d, e). This experimental model revealed that mutagenic NHEJ accounts for >50% of the whole population in shControl, whereas knockdown of BUB1B/BUBR1 expression significantly reduced the event of indels in T24R cells (Fig. 3h, i). Similarly, we observed a decreased ratio of mutagenic NHEJ by the knockdown of BUB1B/BUBR1 expression in JMSU1R cells (Supplementary Figure 2f, 2g). To eliminate the canonical effect of mitotic activity by BUB1B/BUBR1, we performed cell synchronization in the G1 phase by two days of serum starvation, then examined whether the rate of mutagenic DNA repair in response to sgRNA transfection targeting endogenous genomic regions varies in the G1 synchronization with or without BUB1B/BUBR1 knockdown using developed ddPCR experimental model. Interestingly, as shown in Fig. 3j, k, error-prone repair rate was more observed in G1 synchronization than in 10% FBS condition, whereas BUB1B/BUBR1 knockdown resulted in the comparable error-prone repair rates, regardless of the G1 synchronization in the resistant T24R cells. Similar findings were observed in JMSU1R cells as well (Supplementary Figure 2h, i). Collectively, these data suggest that an aberrant BUB1B/BUBR1 expression offers the resistance to DNA-damaging agents by accelerating the mutagenic NHEJ pathway, aside from the canonical activity of BUB1B/BUBR1 previously reported on the G2/M checkpoint.

### Resistance to DSBs by aberrant BUB1B/BUBR1 requires its C-terminal kinase domain and is ATM-dependent

**Fig. 4:**
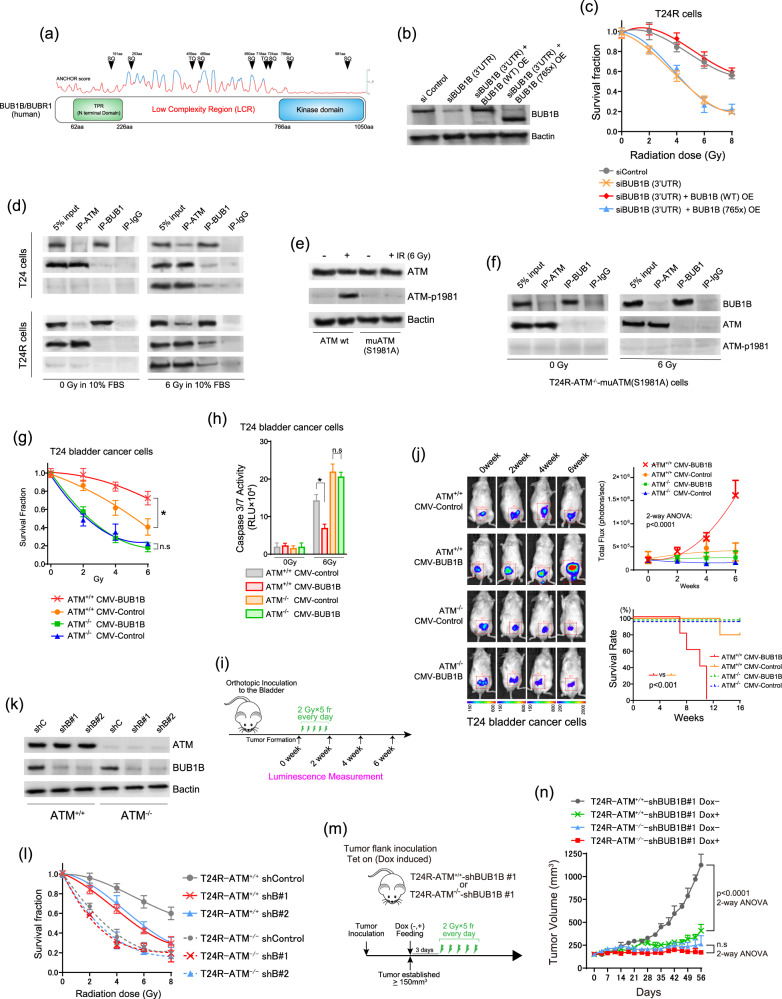
Resistance to DSBs by aberrant BUB1B/BUBR1 requires its C-terminal kinase domain and is ATM-dependent. **a** Schematic representation of BUB1B/BUBR1 exhibiting putative ATM-phosphorylation sites (S/TQ cluster domain). ANCHOR score indicating a low-complexity region (LCR) with its higher score [48] is shown. **b** Immunoblotting of T24R cells cotransfected with or without siBUB1B/BUBR1 (targeting 3'UTR) and indicated overexpression plasmid (BUB1B/BUBR1 wild-type and BUB1B/BUBR1-765x). Three days after the cotransfection, total cell lysates were collected and subjected to the immunoblotting using indicated antibodies. B-actin was loaded as an internal control. **c** Two days after the cotransfection, each cell was treated with IR in the indicated dose, followed by the measurement of cell-viability assay after six days. The inhibitory effect on cell growth by the IR is presented as a relative value (mean ± SD) compared with control (0 Gy) as 100%. **d** Parent T24 and T24R cells were treated with or without IR and incubated for three hours. Nuclear fractions were then collected and immunoprecipitated with antibodies specific to IgG, ATM, and BUB1B/BUBR1, followed by immunoblotting with indicated antibodies. **e** Immunoblotting of T24R-ATM^−/−^ cells transfected with either ATM wild-type or mutated ATM(S1981A) overexpression plasmid. Three days after the transfection, cells were treated with 6 Gy of IR and incubated for three hours. Then, total cell lysates were collected and subjected to immunoblotting using indicated antibodies. B-actin was loaded as an internal control. **f** T24R-ATM^−/−^-muATM(S1981A) cells were treated with or without IR and incubated for three hours. Nuclear fractions were then collected and immunoprecipitated with antibodies specific to IgG, ATM, and BUB1B/BUBR1, followed by immunoblotting with indicated antibodies. **g** Indicated cells were treated with IR in the indicated dose, followed by the measurement of cell-viability assay after six days. The inhibitory effect on cell growth by the IR is presented as a relative value (mean ± SD) compared with control (0 Gy) as 100%. *P < 0.05, two-way ANOVA. **h** Indicated cells were treated with or without 6 Gy of IR. Two days later, caspase 3/7 activity was measured. * indicates *p* < 0.05. **i** Schematic representation of the orthotopic xenograft mouse model. After the orthotopic inoculation, cells were allowed to form the tumor in two weeks, followed by the initiation of IR treatment (0 weeks). Thereafter, the luciferase activity was measured every two weeks (*n* = 5 in each group). **j** Left panel: representative images of the quantitative luminescence measurement for each group in the orthotopic xenograft model following the IR treatment. Right upper panel: quantitative evaluation of the developed tumor in orthotopic xenograft mice. Total flux (photons/sec) in the region of interest (ROI) was recorded every two weeks. * indicates *p* < 0.05 (two-way ANOVA). Right lower panel: Kaplan–Meier curves in the orthotopic mouse model of the indicated cells. A Log-rank test was performed to assess the survival difference. **k** Immunoblotting of shControl and shBUB1B#1,2 in T24R-ATM^+/+^ and T24R-ATM^−/−^ cells. Cells were cultured with 0.15 μg/ml of doxycycline for three days, then subjected to immunoblotting with indicated antibodies. **l** Indicated cells were cultured with 0.15 µg/ml of doxycycline (10% FBS) for 48 h, then treated with IR in the indicated dose. After six days, cell viability was measured. The inhibitory effect on cell growth by the IR is presented as a relative value (mean ± SD) compared with control (0 Gy) as 100%. **m** Schematic of the protocol for the xenograft mouse model. After tumors (T24R-ATM^+/+^-shBUB1B #1 or T24R-ATM^−/−^-shBUB1B #1) developed reaching 150 mm^3^ of tumor volume, mice were divided into four groups (according to dox feeding) with five mice in each group. **n** Tumor growth of T24R-ATM^+/+^-shBUB1B #1 and T24R-ATM^−/−^-shBUB1B #1 cells in the xenograft mouse model (with or without dox feeding) treated with IR treatment. The results are shown as mean ± SD.

It has been reported that BUB1B/BUBR1 is a versatile multidomain protein including putative kinase domain in its C terminus [18] (Fig. 4a). To examine whether the kinase domain in BUB1B/BUBR1 is required for the resistance to DSBs, we conducted BUB1B-765x over-expression that lacks its kinase domain at the C terminus (aa766–1050) following BUB1B knockdown (targeting 3'UTR) in the resistant T24R cells (Fig. 4b). Interestingly, reintroduction of BUB1B-765x expression after BUB1B knockdown (targeting 3'UTR) in T24R cells did not change the sensitivity to IR treatment as compared with the reintroduction of BUB1B-WT (full length) exerting the resistance to IR treatment (Fig. 4c). These data indicate that the response to the IR treatment in the resistant T24R cells is specifically modulated by BUB1B/BUBR1 expression level and the kinase domain of BUB1B/BUBR1 at the C terminus is indispensable for the observed phenotype.

The Ataxia–telangiectasia-mutated kinase (ATM) protein is the initial transducer of DSBs, and several phosphorylated sites in ATM including S1981 have been demonstrated for the dissociation into the active monomer from an inactive homodimeric state after the formation of DSBs [19]. ATM phosphorylates multiple proteins involved in cell-cycle checkpoint control, apoptotic responses, and DNA repair [20]. These proteins contain S/TQ cluster domains specifically recognized by ATM as phosphorylation sites and mainly located on the unfolded region in their native states [21]. As shown in Fig. 4a, BUB1B/BUBR1 has an enrichment of the S/TQ cluster domain in the low structural complexity region (LCR) [18, 22]. Therefore, we examined whether ATM physically binds to BUB1B/BUBR1 with or without IR treatment in BC cells. Co-immunoprecipitation was performed in parent T24 and resistant T24R cells with or without IR treatment. There was no interaction between BUB1B/BUBR1 and ATM without IR treatment in both T24 and T24R cells, whereas phosphorylated ATM (S1981) induced by IR exhibited an interaction with BUB1B/BUBR1 in T24R cells (Fig. 4d). Similar findings were also confirmed in JMSU1R cells (Supplementary Figure 3a). Next, we generated T24R-ATM^−/−^ cells using the CRISPR/Cas9 system followed by single-cell cloning (Supplementary Figure 3b), then conducting the re-introduction of mutated ATM (S1981A). We confirmed that T24R-ATM^−/−^-muATM (S1981A) cells shows no phosphorylation of mutated ATM in aa1981 following IR treatment (Fig. 4e). Importantly, the interaction between ATM and BUB1B/BUBR1 upon DSBs was not observed in T24R-ATM^−/−^-muATM (S1981A) cells, unlike the original T24R cells (Fig. 4f). These data indicate that the cell-cycle-independent overexpression of BUB1B/BUBR1 allows cells to interact with the active monomeric form of ATM (dissociated from homodimeric form by the autophosphorylation of S1981 upon DSBs), which leads to the resistance to DSBs dominantly exploiting NHEJ pathway.

We also generated T24-ATM^−/−^ cells (Supplementary Figure 3c). Overexpression of BUB1B/BUBR1 was performed in parent T24-ATM^+/+^ and T24-ATM^−/−^ cells (Supplementary Figure 3d). Notably, overexpression of BUB1B/BUBR1 conferred resistance to IR and cisplatin treatment in parent T24-ATM^+/+^ cells, whereas T24-ATM^−/−^ cells exhibited hypersensitivity, regardless of the BUB1B/BUBR1 protein expression (Fig. 4g, h, Supplementary Figure 3e). Next, we adopted an in vivo orthotopic xenograft mouse model using T24-ATM^+/+^ and T24-ATM^−/−^ BC cells with or without BUB1B/BUBR1 stable overexpression (Fig. 4i). After the orthotopic inoculation, all the cells including T24-ATM^+/+^ and T24-ATM^−/−^ BC cells with or without BUB1B/BUBR1 stable overexpression uniformly developed tumor with no significant growth difference when no treatment was offered (Supplementary Figure 3f-h). Importantly, overexpression of BUB1B/BUBR1 in T24-ATM^+/+^ cells exhibited tumor growth after the IR treatment, whereas T24-ATM^−/−^ cells did not show tumor growth regardless of the BUB1B/BUBR1 overexpression (Fig. 4j).

To further support these findings, we next used T24R-ATM^−/−^ cells with shBUB1Bs. We confirmed that BUB1B/BUBR1 expression level is not affected by the homozygous deletion of ATM in the resistant T24R cells (Fig. 4k). Following the lentiviral shBUB1B infection, we examined sensitivity to IR treatment in these cells. Knockdown of BUB1B/BUBR1 reverted the resistance to IR treatment in T24R-ATM^+/+^ cells, whereas T24R-ATM^−/−^ cells showed hypersensitivity, regardless of the BUB1B/BUBR1 knockdown (Fig. 4l). We further conducted an in vivo xenograft mouse model using T24R-ATM^+/+^ and T24R-ATM^−/−^ BC cells with or without BUB1B/BUBR1 knockdown (Fig. 4m). Importantly, knockdown of BUB1B/BUBR1 in the resistant T24R-ATM^+/+^ cells abrogated the tumor growth after the IR treatment, whereas T24R-ATM^−/−^ cells did not grow, regardless of the BUB1B/BUBR1 knockdown after the IR treatment (Fig. 4n), consistently suggesting the crucial interaction between ATM and BUB1B/BUBR1 in the context of the resistance to DNA-damaging agents.

### Therapeutic implication of the ATM inhibition and publicly available dataset analysis in BC patients

**Fig. 5:**
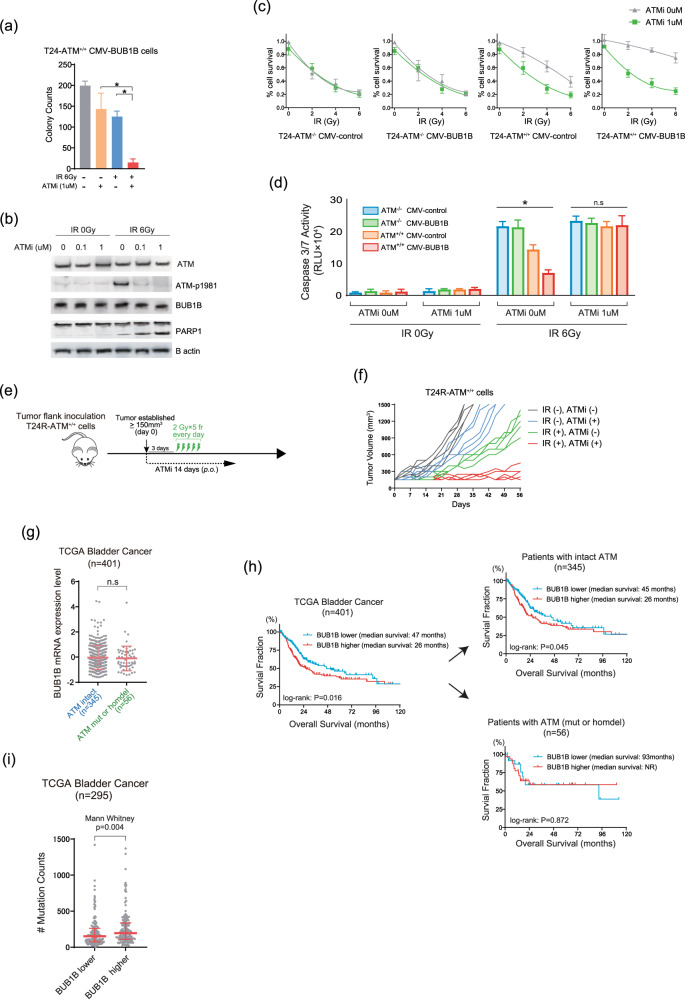
Therapeutic implication of the ATM Inhibition and publicly available dataset analysis in BC patients. **a** The number of colonies of clonogenic survival assay counted in five random fields in 21 days. T24-ATM^+/+^ CMV-BUB1B overexpression cells were plated to 6 well plates and treated with or without 1 µM of ATM inhibitor (AZD0156). Twenty-four hours later, IR (0 Gy or 6 Gy) treatment was administered. The medium was changed every three days. The results are shown as mean ± SD. * indicates *p* < 0.05. **b** Immunoblotting of T24-ATM^+/+^ CMV-BUB1B-overexpression cells. Cells were treated with the ATM inhibitor 24 h before the administration of IR treatment. Twenty-four hours later, cells were collected and subjected to immunoblotting with indicated antibodies. B-actin was loaded as an internal control. **c** Cells were treated with or without ATM inhibitor 24 h before administering IR in indicated dose at day 0, followed by the measurement of cell viability assay after six days. The inhibitory effect on cell growth by the IR is presented as a relative value (mean ± SD) compared with control (0 Gy) as 100%. **d** Cells were treated with or without the ATM inhibitor 24 h before administering IR in the indicated dose. Two days later, caspase 3/7 activity was measured. * indicates *p* < 0.05 (one-way ANOVA). **e** Schematic of the protocol for the xenograft mouse model. After tumors (T24R-ATM^+/+^ cells) developed reaching 150 mm^3^ of tumor volume, mice were divided into four groups with five mice in each group. AZD0156 (10 mg/kg daily Q.D.) was given orally (P.O.) for two weeks. IR treatment (2 Gy x 5fr: 10 Gy) was started three days after the initiation of AZD0156. **f** Tumor growth of T24R-ATM^+/+^ cells in the xenograft mouse model (with or without AZD0156 and IR treatment). The results are shown as mean ± SD. **g** BUB1B/BUBR1 mRNA expression level according to the ATM mutation status in the TCGA BC dataset (*n* = 401) [10]. **h** Kaplan–Meier curves in the TCGA BC data set according to the BUB1B mRNA-expression level. Patients were then stratified according to the ATM mutation status. In all the analyses, patients were divided by the median cut-off of BUB1B mRNA expression level. A log-rank test was carried out to examine the survival difference. **i** Mutation counts according to the BUB1B mRNA expression level in the TCGA BC dataset. All the data were downloaded from the cBio Cancer Genomics Portal (cBioPortal; www.cbioportal.org). Patients were divided by the median cutoff of BUB1B mRNA-expression level. Mann–Whitney U test was conducted to assess the difference between the two groups.

There have been a number of studies demonstrating that the blockage of ATM activity confers an increased vulnerability to DSBs in various types of cancers [20, 23, 24]. Therefore, we examined whether the ATM inhibition using AZD0156, a potent and selective inhibitor of ATM kinase [25, 26], resensitizes cells with aberrant BUB1B/BUBR1 expression to IR and cisplatin treatment. We first tested AZD0156 to T24-ATM^+/+^ with BUB1B/BUBR1 stable-overexpression cells in combination with cisplatin treatment (Supplementary Figure 4a). In agreement with the result of T24-ATM^−/−^ BC cells showing high sensitivity to DSBs, regardless of BUB1B/BUBR1 expression level, ATM inhibition by AZD0156, substantially abrogated the resistance to IR treatment in T24-ATM^+/+^ with BUB1B/BUBR1 stable-overexpression cells (Fig. 5a). Immunoblotting exhibited that AZD0156 hinders upregulation of phosphorylation of ATM (S1981) induced by IR treatment without altering BUB1B/BUBR1 protein expression, which results in the increased cleaved PARP1 expression (Fig. 5b). Importantly, inhibition of ATM activity had no synergistic effect on IR treatment in ATM-null cells that inherently exhibits prominent sensitivity, whereas ATM^+/+^ cells showed a robust sensitization to IR treatment by AZD0156 regardless of BUB1B/BUBR1 overexpression into the level of sensitivity in ATM-null cells (Fig. 5c). This was also supported by the result of caspase3/7 activity in which the apoptosis following IR treatment was less induced in BUB1B/BUBR1-overexpression cells than the control cells in ATM^+/+^ cells, and ATM kinase inhibition produced as much apoptosis with or without BUB1B/BUBR1-overexpression as ATM-null cells (Fig. 5d). We further conducted in vivo experiments using the CRT-resistant T24R-ATM^+/+^ cells that show an increased BUB1B/BUBR1 expression level compared with the parent T24 cells. AZD0156 (10 mg/kg daily Q.D.) was given orally (P.O.) for two weeks, and IR treatment (2 Gy x 5fr: 10 Gy) was started three days after the initiation of AZD0156 (Fig. 5e). Importantly, monotherapy with AZD0156 offered a modest effect for the inhibition of tumor growth in T24R-ATM^+/+^ cells, whereas the combination of AZD0156 and IR treatment showed a durable suppression of tumor growth (Fig. 5f). These data indicate the potential utility of ATM kinase inhibition by AZD0156 in CRT-resistant cells with increased BUB1B/BUBR1 expression level.

We next explored publicly available datasets. Lee’s dataset showed that patients with higher BUB1B/BUBR1 expression level have significantly shorter overall survival (Supplementary Figure 4b) [27]. We then analyzed the association of BUB1B/BUBR1 expression and ATM mutations in the TCGA BC dataset [10]. Comparable BUB1B/BUBR1 mRNA-expression level was seen between ATM-intact and ATM-mutation patients (Fig. 5g), which was consistent with the result that knockout of ATM does not affect BUB1B/BUBR1 expression level as shown in Fig. 4k. In line with the result from Lee’s cohort, higher BUB1B/BUBR1-expression level was significantly correlated with shorter overall survival (Fig. 5h). Of note, when stratified by the presence of ATM mutations, shorter overall survival in patients with higher BUB1B/BUBR1-expression level was observed in patients with intact ATM, but not in patients who had ATM mutation. A similar finding was observed in TCGA lung adenocarcinoma, in which higher BUB1B/BUBR1-expression was significantly correlated with shorter overall survival in patients with intact ATM, whereas no survival difference was observed according to BUB1B/BUBR1-expression level in patients with ATM mutation (Supplementary Figure 4c) [28]. We further confirmed that higher BUB1B/BUBR1-expression level is consistently associated with poor clinical outcomes in various cancers (Supplementary Figure 4d) [29, 30]. There seems to be no difference in overall survival between patients with and without ATM mutation in BC, lung adenocarcinoma, kidney clear-cell carcinoma, and hepatocellular carcinoma (Supplementary Figure 4e). Based on the results from the experiments that indicate mutagenic NHEJ with increased BUB1B/BUBR1 expression, we hypothesized that BC tumor with aberrant BUB1B/BUBR1 expression level harbors higher mutation counts. In the TCGA BC dataset, patients with higher BUB1B mRNA expression levels had significantly increased mutation count compared with patients with lower BUB1B mRNA expression levels (Fig. 5i).

### The transcription factor FOXM1 activates the BUB1B/BUBR1 expression

**Fig. 6:**
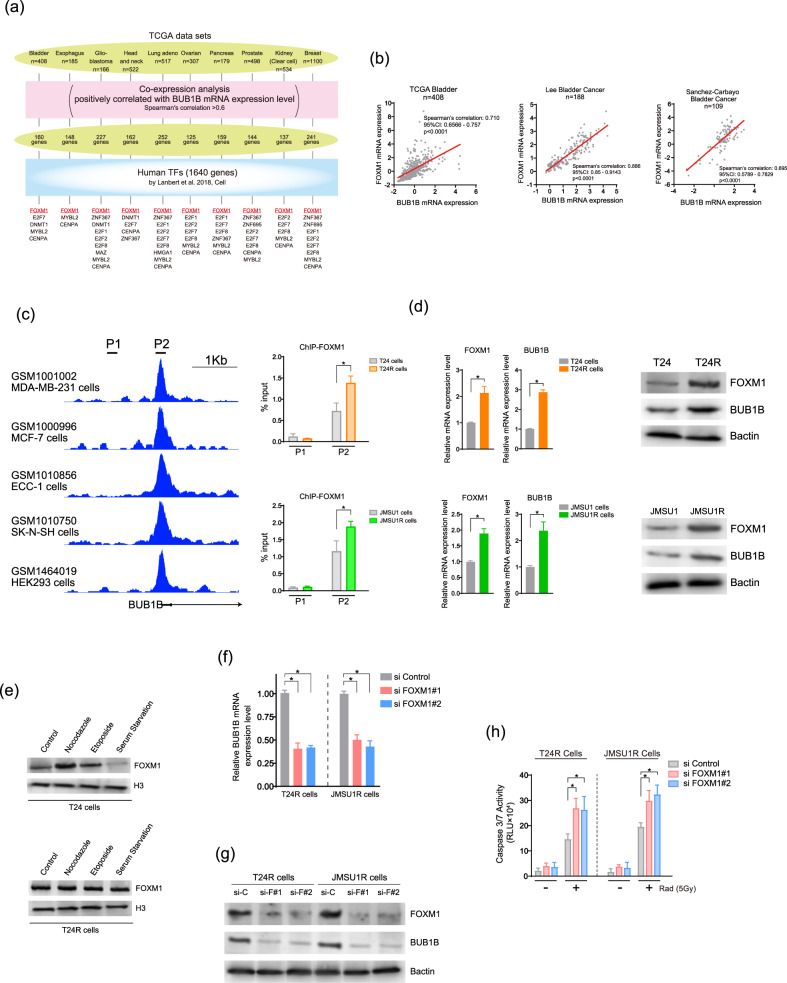
The transcription factor FOXM1 activates the BUB1B/BUBR1 expression. **a** The putative transcription factors that regulate the expression of BUB1B/BUBR1. Genes positively correlated with BUB1B/BUBR1-expression level (Spearman’s correlation coefficient > 0.6) in TCGA datasets among various cancer types were compared with the putative human transcription factors [33]. **b** The correlation between BUB1B/BUBR1 and FOXM1 mRNA-expression levels in the publicly available BC datasets [10, 27, 34]. **c** Left panel: ChIP-seq datasets of FOXM1 [49–51]. The chromatin status in the vicinity of transcription-start site (TSS) and primers designed on BUB1B/BUBR1 are shown. Right panel: Quantitative PCR of chromatin immunoprecipitation (ChIP) of FOXM1 was performed in indicated BC cells. Data are shown as mean ± SD. * indicates *p* < 0.05. **d** Left panel: The quantitative PCR for the mRNA-expression level of FOXM1 and BUB1B/BUBR1 in indicated BC cells. Data are shown as mean ± SD. * indicates *p* < 0.05. Right panel: Immunoblotting of FOXM1 and BUB1B/BUBR1 in indicated BC cells. B-actin was loaded as an internal control. **e** Immunoblotting in T24 and T24R cell lines. Cell-cycle synchronization was performed by serum starvation in 0.1% FBS (G1 phase), 0.5 µM of etoposide (S phase), and 1 µM of nocodazole (G2/M phase) for 36 h. Then, nuclear fractions were subjected to immunoblotting with the indicated antibodies. Histone 3 was loaded as an internal control. **f** The quantitative PCR in T24R and JMSU1R BC cells. Cells were transfected with indicated siRNAs and then collected for the analysis 48 h after the transfection. Data are shown as mean ± SD. * indicates *p* < 0.05. **g** Immunoblotting of FOXM1 and BUB1B/BUBR1 in T24R and JMSU1R BC cells. Cells were transfected with indicated siRNAs and then collected for the analysis 72 h after the transfection. **h** Cells were transfected with indicated siRNAs and incubated for 48 h. Then, cells were treated with or without 5 Gy of IR. Caspase 3/7 activity was measured 48 h after the administration of IR treatment. Data are shown as mean ± SD. * indicates *p* < 0.05.

There have been several studies that have focused on transcriptional activation of BUB1B/BUBR1 in cancers [31, 32]. Mutations and amplification of BUB1B/BUBR1 seem to be rare in BC patients (Supplementary Figure 5a). Hence, we speculated that transcriptional regulation might largely contribute to the overexpression of BUB1B/BUBR1. To identify the putative transcription factors that regulate the expression of BUB1B/BUBR1, we investigated genes positively correlated with BUB1B/BUBR1 expression level (Spearman’s correlation coefficient >0.6) in TCGA datasets among various cancer types, followed by the extraction of putative human transcription factors [33] from these positively correlated genes (Fig. 6a). Of all the datasets included in the analysis, FOXM1 was identified as a putative transcription factor that regulates the BUB1B/BUBR1 expression (Supplementary Figure 5b). We further validated the positive correlation of the expression between BUB1B/BUBR1 and FOXM1 in other BC datasets (Fig. 6b) [10, 27, 34].

We sought to characterize the FOXM1-binding site on the BUB1B/BUBR1 promoter region from publicly available datasets of FOXM1 ChIP-Seq (Fig. 6c). We identified enrichment of FOXM1 in the BUB1B/BUBR1 promotor region (P2) compared with a negative-control region (P1). Furthermore, this enrichment was more pronounced in T24R and JMSU1R cells than in parent T24 and JMSU1 BC cell lines (Fig. 6c). These data assist the hypothesis that aberrant BUB1B/BUBR1 expression is at least partially attributed to transcriptional activation by the increased FOXM1 expression level. Indeed, increased mRNA and protein expression of FOXM1 were validated in T24R and JMSU1R cells compared with the parent T24 and JMSU1 cell lines (Fig. 6d). We further confirmed that other putative FOXM1-regulated genes [35, 36], including CCNB1, CDC25B, and AURKB, were also upregulated in T24R and JMSU1R cells compared with the parent T24 and JMSU1 cell lines (Supplementary Figure 6a). Cell synchronization was performed in both T24 and T24R cells to assess whether FOXM1 protein-expression level across the cell-cycle phases differs between those cells. As shown in Fig. 6e, T24R cells seemed to have constitutively upregulated FOXM1 protein expression across the cell cycle phases compared with the parent T24 cells, being in line with the findings of BUB1B/BUBR1 shown in Fig. 2g.

The ATM mutation status did not affect FOXM1 mRNA-expression level in the TCGA BC dataset, suggesting that increased BUB1B/BUBR1 expression by FOXM1 and ATM status was independently determined in BC patients (Supplementary Figure 6b). We performed siFOXM1 knockdown in T24R and JMSU1R cells and confirmed that knockdown of FOXM1 leads to the decreased BUB1B/BUBR1 expression in mRNA level (Fig. 6f, Supplementary Figure 6c) as well as protein-expression level (Fig. 6g). Finally, knockdown of FOXM1 in T24R and JMSU1R cells sensitized IR treatment with increased caspase 3/7 activity in siFOXM1 compared with negative control, indicating the abrogation of downstream targets, including BUB1B/BUBR1 (Fig. 6h). Altogether, these data suggest that FOXM1 can transcriptionally activate BUB1B/BUBR1 expression by directly binding to its promoter region.

## Discussion

BUB1B/BUBR1 constitutes the mitotic-checkpoint complex (MCC) with Bub3, Mad2, and Cdc20, which inhibits the anaphase-promoting complex/cyclosome (APC/C) and in turns controls mitotic phases [37]. In addition to those canonical functions, the results from the current study suggest that a constitutive upregulation of BUB1B/BUBR1 throughout the cell-cycle phases in CRT-recurrent tumor offers a redundant function to repair DSBs, which dominantly exploits mutagenic NHEJ rather than precise NHEJ or HR, leading to the CRT-resistant clones harboring accumulated mutations.

There have been several studies proposing that BUB1B/BUBR1 participates in DNA repair, whereas it’s biological mechanism is still controversial [38]. For instance, Fang et al. showed that BUB1B/BUBR1^+/−^ murine fibroblasts (MEFs) have a higher survival rate in response to DSBs by the treatment using doxorubicin compared with wild-type BUB1B/BUBR1^+/+^ MEFs [39]. In contrast, Thompson et al. exhibited that BUB1B/BUBR1 negates caspase2-dependent apoptosis in response to IR by outcompeting recruitment of RAIDD to the death domain of PIDDosome [40]. Our findings supported the hypothesis that increased BUB1B/BUBR1 expression is associated with resistance to IR. Of note, we identified that ATM, the initial transducer kinase for DNA damage, interacts with BUB1B/BUBR1 after IR treatment, further indicating the redundant function of BUB1B/BUBR1 underlying the biological process of DNA repair, especially in mutagenic NHEJ.

Accumulated evidence suggests that tumors with indels in ATM have hypersensitivity to DNA toxic modalities [41]. Hence, the recent studies have prevalently focused on the biological mechanism in tumor cells devoid of functional ATM, such as synthetic lethal approach using poly-ADP ribose polymerase (PARP) inhibitors [42–44]. Nevertheless, the prognostic impact of ATM mutations seems to be limited in the analysis of TCGA datasets including BC patients (Supplementary Figure 4e), and the majority of BC patients (86%: 345 out of 401) had no mutation in ATM, which implies the importance of intact ATM as a therapeutic target. This is supported by the report from advanced metastatic colorectal cancer, in which patients with intact ATM (85%: 192 of 227 patients) had significantly poor overall survival compared with those with ATM mutations (15%: 35 of 227 patients) [45]. Several studies have reported that ATM inhibitors could reverse the resistance to DSBs induced by IR and cisplatin [23, 25, 26]. Given the findings that the resistance to DSBs by aberrant BUB1B/BUBR1-expression level is attributed to intact ATM, it is plausible that an increased BUB1B/BUBR1 expression predicts who would benefit from ATM inhibitor in combination with DNA damaging agents.

## Supplementary information

Supplementary Methods

Supplementary Figure 1

Supplementary Figure 2

Supplementary Figure 3

Supplementary Figure 4

Supplementary Figure 5

Supplementary Figure 6

Legends of Supplementary Figures

Supplementary Table 1

Supplementary Table 2

Supplementary Materials
