# Supplementary material for: Increased BUB1B/BUBR1 expression contributes to aberrant DNA repair activity leading to resistance to DNA-damaging agents
Source: Oncogene. 2021 Sep 20;40(43):6210–22. doi: 10.1038/s41388-021-02021-y (PMC8553621; doi:10.1038/s41388-021-02021-y)
Supplement: Supplementary file 1 — Supplementary Methods [file 41388_2021_2021_MOESM1_ESM.docx]

**Supplementary Methods**

*Cell Lines, Proliferation Assay, Caspase-3/7 Assay*

The BC cell lines were characterized as previously reported [6, 7]. JMSU1 cells were kindly provided from Jichi medical school in Japan (Tochigi)[8]. 253JBV cells were provided from MD Anderson Cancer Center [9], and all the other cell lines in the study were obtained from the American Type Culture Collection (ATCC). The authentication was obtained by Human STR Profiling Cell Authentication Service, and the mycoplasma test was tested in all cell lines. These cells were maintained in 10% fetal bovine serum supplemented with 2mM of L-glutamine at 37 °C in 5% CO2. Cells treated in individual experiments were assessed for cell viability using Cell Titer-Fluor Assay and Caspase-Glo® 3/7 Assay (Promega, Madison, WI) following the manufacturer’s protocol.

*Tandem mass tag (TMT)-labeling Quantification for Mass Spectrometry (MS)*

TMT 10 plex Isobaric Label Reagent Set, Pierce Quantitative Colorimetric Peptide Assay, was purchased from Thermo Fisher Science. Samples were reduced by 10mM Triethylammonium bicarbonate buffer (TEAB: 1.0 M, pH 8.5±0.1) at 56°C for 1 hour, followed by trypsinization at a ration of 1:50 for 37°C overnight. After lyophilizing the extracted peptides to near dryness, 100µg of peptides were diluted for a final volume of 100uL at 50mM. Immediately after the equilibration of TMT Label Reagents to room temperature, 41μL of anhydrous acetonitrile was added to each sample tube, followed by 5 minutes incubation with an occasional vortex. Then, samples were mixed with TMT Label Reagents for 1 hour at room temperature. Eight μL of 5% hydroxylamine was added to quench the reaction, and samples were combined into one tube. High-performance liquid chromatography (HPLC) was performed to fractionate the sample with six components. For nanoLC-MS/MS Analysis, a Dionex Ultimate 3000 Nano LC system coupled with an Orbitrap Q Exactive mass spectrometer (Thermo Fisher Scientific, USA) with an ESI nanospray source was used with a setting as follows (Nanocolumn: 100 μm×10 cm in-house made column packed with a reversed-phase ReproSil-Pur C18-AQ resin, loaded sample volume: 5 μL, Total flow rate: 600 nL/min, MS resolution:70000 at 400 m/z , MS precursor m/z range: 300.0-1650.0). For data analysis, the six raw MS files were analyzed and searched against the human protein database based on the species of the samples using Maxquant (ver.1.5.6.5). Gene Ontology analysis was performed using DAVID bioinformatics resources (https://david.ncifcrf.gov). The heat map was created using GENE-E (www.broadinstitute.org/cancer/software/GENE-E/).

*Soft Agar Colony Formation Assay*

Fifty thousand live cells (T24R and JMSU1R cells with indicated shRNAs) were mixed with 0.3% top-agar and plated onto a 0.6% base-agar in six-well plates triplicates. The cells were treated with 1µM of cisplatin for three weeks changing the FBS medium with 0.15 µg/ml doxycycline every three days, followed by 0.1% iodonitrotetrazolium chloride (INT) staining overnight. The plates were photographed, and the colony numbers were counted by Gelcount (Oxford oOptonix, Abington, UK).

*Cell Cycle Analysis*

Cells were cultured with indicated drugs for 72 hours, and one million cells were collected, followed by washing with HBSS and fixing with ice-cold 70% ethanol overnight. Then, the cells were re-washed with HBSS, stained with 200 μL of PI/RNase reagent (Millipore, Darmstadt, Germany) for 30 min, and the distribution of cell cycle was analyzed by the BD FACSAria cell sorter (BD Biosciences, New Jersey, USA). Data obtained was analyzed with FlowJo Software (Treestar, Ashland, OR).

*Quantitative PCR*

RNA was isolated using TRIzol (Invitrogen, Carlsbad, CA) and Direct-zol RNA Prep Plus (Zymo Research, Irvine, CA) according to the manufacturer’s protocol followed by quantification using a NanoDrop spectrophotometer, and 100-500 ng of RNA was reverse-transcribed using a SuperScript IV VILO Master Mix (Invitrogen, Carlsbad, CA, United States). The primers used are listed in Supplementary Materials. Quantitative PCR was performed on an ABI Quant Studio 5 detector. Product formation was detected by incorporation of SYBR Green I using ROX as a passive reference. The expression data were normalized with GAPDH in each sample. Experiments were independently repeated and analyzed three times.

Immunofluorescence staining

BC cells with indicated shRNAs were plated onto collagen-coated 24 well culture dish for two days, followed by fixation in 4% paraformaldehyde (PFA) at room temperature for 10 min, then washed three times with HBSS, and incubated with 0.1 M glycine for 10 min to quench PFA autofluorescence. After washing again, cells were permeabilized and stained with a solution of 0.1% Triton X–100 and HBSS + BSA 1%, containing primary antibodies at a ratio of 1:250. Cells were then washed and incubated for 60 min with appropriate secondary–fluorescent antibodies (Alexa Fluor 488 anti-rabbit secondary antibody and Alexa Fluor 568 anti-mouse secondary antibodies purchased from Invitrogen, Life Technologies, Carlsbad, CA, United States). After washes, the nucleus was stained with DAPI reagent (Invitrogen, Life Technologies, Carlsbad, CA, United States) overnight at -20 degrees. The images were obtained and analyzed using the BZ-X800 analyzer (Keyence, Osaka, Japan).

*RNA Interference, DNA Transfection, and Lentiviral Transduction*

The sequences of shRNAs used are listed in Supplementary Materials. Mammalian Gene Collection human BUB1B/BUBR1 sequence-verified cDNA (Catalog ID: MHS6278-202832130) was purchased from Dharmacon (Horizon Discovery). The plasmid of human ATM sequence-verified cDNA with CMV promotor (Catalog ID: OHS5898-224629547) was purchased from Dharmacon (Horizon Discovery). A point mutation of ATM-S1981A (Ser: AGC to Ala: GCC) was generated using PrimeSTAR® Mutagenesis Basal Kit (TAKARA BIO, Japan). Individual shRNAs were designed using Enhanced Direct (rnai.co.jp/lsci/e-sidirect.html) for licensees considering mismatch potential >0.3 and the longest common factor (LCF) < 9. siRNA transfections targeting BUB1B/BUBR1 and FOXM1 were purchased from Dharmacon (Horizon Discovery: listed in Supplementary Materials) and performed using Lipofectamine RNAiMAX (Invitrogen, Carlsbad, CA, United States). Twenty-four hours before transfection, cells were seeded onto six-well plates. The cells were transfected with 50 nM siRNA as described in the manufacturer’s protocol and maintained for 48 hours, followed by the designed experiments. The sequence of siBUB1B/BUBR1 targeting 3’UTR is provided in Supplementary Materials. For overexpression of BUBR1-765x, GENEART and GENEOPTIMIZER services (Thermo Scientific, Waltham, MA) were applied for the design of the plasmid (CDS presented in Supplementary Materials), and co-transfection with siBUB1B(3’UTR) was performed using DharmaFECT Duo Transfection Reagent (Horizon Discovery). For lentiviral transduction, pLKO-Teton-puro (Addgene plasmid ID: #21915) and pLenti-CMyc-DDK-IRES-Puro for overexpression of BUB1B/BUBR1 (Origene plasmid ID: PS100069), pHAGE PGK-GFP-IRES-LUC-W (Addgene plasmid ID: #46793), and TRE-KRAB-dCas9-IRES-GFP (Addgene plasmid ID: #85556) vectors were transfected with psPAX2 packaging and pMD2.G envelope plasmid to HEK293FT cells using Lipofectamine 3000 (Invitrogen, Carlsbad, CA, United States) for two days. Thereafter, indicated cells were infected with viral supernatants (filtered through a 0.45-um filter) in the presence of 8 μg/mL polybrene. For overexpression of BUBR1-765x, GENEART and GENEOPTIMIZER services (Thermo Scientific, Waltham, MA) were applied to design the plasmid (CDS presented in Supplementary Materials). For shRNAs, a spin-infection protocol was applied using six-well plates at 2,700 rpm for 60 min (Heraeus Multifuge X1 Centrifuge Series, Thermo Scientific, Waltham, MA), followed by incubation at 37 °C. The next day, the medium was changed to a fresh medium, and the cells transduced with the virus were incubated for three days, followed by selection using puromycin (1–1.5 ng/mL).

*Flow Cytometry to Detect Mutagenic NHEJ and HR Repair*

Lentiviral infection of TRE-KRAB-dCas9-IRES-GFP (Addgene plasmid ID: #85556) was performed in 293T cells and T24R cells to stably express dCas9 and integrate DNA sequence (IRES-GFP). gRNA targeting GFP with a U6 promoter was cloned into the pMA-T backbone vector (pMA-T-U6-sgGFP). Knock-in donor DNA for HR repair was designed to express mCherry with both homology arms (>500 bp) across the sgGFP targeted site (1838 bp: Supplementary Materials) and cloned into pMK-RQ backbone vector. Then, the knock-in donor dsDNA was amplified by PCR using Platinum SuperFi DNA Polymerase (Thermo Scientific, Waltham, MA) (primers listed in Supplementary Materials), followed by digestion of phosphorylated strand to generate knock-in donor ssDNA using Guide-it Long ssDNA Strandase (Takara-bio, Kusatsu, Shiga). For the transfection, pMA-T-U6-sgGFP and knock-in donor ssDNA were simultaneously transfected to the indicated cells stably expressing dCas9 in reverse transfection protocol using lipofectamine 3000 according to manufacture’s protocol. After 72 hours, cells were collected and analyzed by the BD FACSAria cell sorter (BD Biosciences, New Jersey, USA) to calculate the change of proportion in GFP positive and mCherry positive cells. Experiments were independently repeated and analyzed three times.

*CRISPR RNP transfection and single-cell clonal isolation for the generation of ATM^-/-^ cell line*

Cells were plated onto twelve well plates at 30% confluency. We used the Truguide CRISPR sgRNA system (Thermo Scientific, Waltham, MA). In short, crRNA (targeting ATM, AAVS, RBM20: Supplementary Materials) and tracrRNA (100 μM) containing 10 μL of 5X Annealing Buffer were mixed in nuclease-free water for a final crRNA:tracrRNA duplex (sgRNA) concentration of 20 μM, followed by annealing in a thermal cycler. The next day, the CRISPR ribonucleoprotein (RNP) complex that consisted of sgRNA and Cas9 protein (TrueCut Cas9 Protein v2) was transfected using the CRISPRMAX transfection reagent (Thermo Scientific, Waltham, MA) according to the manufacture’s protocol. In brief, sgRNA (250ng) and cas9 protein (2 µg) were added to the Opti-MEM medium, followed by adding 3µl of Cas9 Plus reagent with a total volume of 50 µl (tube 1). Tube 2 was mixed in 50 µl Opti-MEM medium with 3 µl of Lipofectamine CRISPRMAX reagent (Thermo Scientific, Waltham, MA). The mixtures were incubated at room temperature for five minutes and then mixed carefully (tube 1 was transferred to tube 2). Ten minutes after the mixing for forming Cas9 RNP and Lipofectamine CRISPRMAX complexes, the mixture was added to the cells.

For single-cell clonal isolation for the generation of T24 ATM^-/-^ cells, the efficiency of genome modification was confirmed using the GeneArt Genomic Cleavage Detection kit following the manufacture’s protocol. To obtain single-cell clones from the total population of sgATM-transfected T24 cells, serial dilution protocol on poly-L-lysine-coated 96-well plate was performed to make 0.5 cell/well density. After the clonal expansion, DNA was extracted from each clone. PCR amplicon was obtained across the sgRNA targeted site (primers listed in Supplementary Materials) in each clone, and the amplicons were inserted into the pCR4-Blunt TOPO Vector kit (Thermo Scientific, Waltham, MA) followed by picking-up five colonies in each clone. Sanger sequencing was performed to determine the sequence at the sgATM targeting locus.

*Digital Droplet PCR (ddPCR)*

CRISPR ribonucleoprotein complexes targeting genomic AAVS and RBM 20 regions were transfected to the indicated cells using CRISPRMAX transfection reagent (Thermo Scientific, Waltham, MA). After 48 hours, genomic DNA was extracted using QIAamp DNA Mini Kit (Qiagen, ‎Venlo, Netherlands). Primers and probes used in ddPCR are listed in Supplementary Materials. Probes were designed on sgRNA targeted sites for FAM reporters and reference sites for HEX reporters. QX200 system (Bio-Rad, ‎Hercules, CA, United States) was utilized in the study. The reactions were performed in 20 ml volume that consisted of 10 mL of 2x ddPCR Supermix for probes (No dUTP; Bio-Rad), primers (900 nmol/L), probes (250 nmol/L) and 50ng of the genomic DNA sample. Each reaction mix was converted to droplets with the QX200 droplet generator. Droplet-partitioned samples were then transferred to a 96-well plate, sealed, and cycled in a C1000 Touch thermal cycler (Bio-Rad) under the following cycling protocol: 95℃ for 10 minutes, followed by 40 cycles of 94℃ for 30 seconds, 60℃ for 60 seconds, and a 10-minute incubation at 98℃. The cycled plates were then read on a Bio-Rad QX200 droplet reader. At least two negative control wells with no genomic DNA template were included in every run. The data analysis was conducted with QuantaSoft droplet reader software v1.7.4 (Bio-Rad, ‎Hercules, CA, United States).

*Immunoblotting, Cell Fractionation, and Co-Immunoprecipitation (CO-IP)*

Whole-cell lysates were collected and lysed in radioimmunoprecipitation assay (RIPA) lysis buffer with proteinase inhibitor mixture (Thermo Scientific, Waltham, MA) and sonicated using a Bioruptor Standard. For cellular protein fractionation, hypotonic lysis buffer (50 mM Hepes-NaOH, pH 7.5, 10% glycerol, 0.5% Nonidet P-40, 0.25% Triton X-100, proteinase inhibitor mixture) was used for extracting cytoplasmic proteins. Nuclear pellets were washed with cold PBS once and dissolved in high-salt nuclear extraction buffer (0.1% SDS, 10 mM Tris·HCl, 150 mM NaCl, 0.1% Triton-X, proteinase inhibitor mixture) and sonicated using a Bioruptor Standard, followed by gentle agitation for 30 min at 4 °C. After centrifugation at 13,200 rpm for 10 min, the supernatant was collected as nuclear fractions. Proteins were subjected to NuPAGE Bis-Tris Gels or NuPAGE Tris-Acetate Gels before being transferred onto the PVDF membrane (Millipore, Darmstadt, Germany). The list of antibodies is provided in Supplementary Materials. Detection of the protein was performed using the Fusion FX imaging system, and Fusion-Capt Advance analyzing system was used to quantify the protein expression levels. For co-immunoprecipitation (CO-IP), nuclear fraction was collected and diluted using dilution buffer (20 mM Tris-HCl pH 8.0, 1.5 mM MgCl2, 0.2 mM EDTA, 0.5 % NP-40). Then, MgCl and DNase (NEB) were added for a final concentration of 3 mM for Mg2+ and 20 U/ml for DNase, followed by 37 c incubation for 30 min. Dynabeads Protein G (Life Technologies) (30 µl) was used to pre-clear for 60 min at 4 c with gentle rotation. Five percent of lysate was kept as input, and the rest was incubated with 5 µg of primary antibodies (BUB1B/BUBR1, ATM, Mouse-IgG: SC-2025) overnight. The next day, 50 µl of Dynabeads Protein G was added and incubated for two hours at 4 c with gentle rotation. After washing beads by washing buffer (10mM Tris pH 7.4, 1mM EDTA, 1mM EGTA pH 8.0, 150mM NaCl, 1% Triton X-100, 0.2mM sodium orthovanadate, Protease inhibitor cocktail), precipitated protein was eluted by heating in 50 µL of 2 x SDS loading buffer without DTT for 10 min at 50°C.

*Chromatin Immunoprecipitation*

Cells (7.5 × 10^6^) at room temperature were cross-linked for 10 min with 1% paraformaldehyde, and the reaction was terminated by the addition of 1 mL of 1.25 M glycine for 5 min, followed by extracting the nuclear fraction using the hypotonic lysis buffer described in the cell fractionation protocol. Cross-linked chromatin was sonicated to an average fragment size of 200–300 bp in 0.2% SDS shearing buffer using a BIORUPTOR II Sonicator. Then, sonicated chromatin was centrifuged for 5 min at 13,000 rpm and diluted to 0.1% SDS concentration. After pre-clearing with Dynabeads Protein G (Life Technologies, Carlsbad, CA) for one hour, chromatin–protein complexes were immunoprecipitated with 5 μg of antibodies overnight at 4 °C. The next day, Dynabeads Protein G was added for 2 h, and beads were washed with the following buffers: low-salt wash buffer (20 mM Tris·HCl, pH 8.0, 150 mM NaCl, 0.1% SDS, 1% Triton X-100, 2 mM EDTA), high-salt wash buffer (20 mM Tris·HCl, pH 8.0, 500 mM NaCl, 0.1% SDS, 1% Triton X-100, 2 mM EDTA), LiCl wash buffer (10 mM Tris·HCl, pH 8.0, 250 mM LiCl, 1% IGEPAL CA-630, 1% sodium deoxycholate, 1 mM EDTA), and TE buffer. Precipitated chromatin was then eluted from the beads in 300 μL elution buffer (1% SDS, 0.1 M NaHCO3) for one hr at room temperature followed by de–cross-linking at 65 °C overnight. After RNase A and proteinase K treatment, ChIP and input DNA were extracted by phenol-chloroform extraction. Fragment sizes (200–300 bp) were evaluated by a High Sensitivity DNA Kit (Agilent Technologies, Santa Clara, CA) on an Agilent 2100 Bioanalyzer. The specific enrichment was analyzed by qPCR and percent input calculation. Primers used in ChIP-qPCR are listed in Supplementary Materials.

*In vivo experiments*

NOD/SCID mice (NOD.CB17-Prkdcscid/J: Charles River, Wilmington, MA) were anesthetized using isoflurane. Tumor cells were inoculated under sterile conditions. For the subcutaneous BC xenograft model, cells were prepared at 2.5 million cells in 50 µl PBS and mixed with 50 µl of Matrigel (Corning Matrigel Matrix High Concentration, Corning, NY) in a total of 100 µl cell suspension followed by the subcutaneous inoculation. After the tumor developed reaching 150 mm^3^ of tumor volume (calculated by use of the modified ellipsoid formula; i.e., length × width^2^/2), mice were randomized into groups according to the administration of radiation (2Gy: 5 fractions daily) and 0.1% doxycycline feeding in each group. For ATM inhibition, AZD0156 (10 mg/kg daily *Q.D.*) was given orally *(P.O.*) for two weeks. In short, 100 µl of stock solution for AZD0156 (2mg/ml in molecular biology grade ethanol) was mixed with an equal volume of polyethylene glycol-300 and ddH2O (a total of 300µl solution), then administrated to each mouse by oral gavage. Tumors were manually measured every week, and any mice were sacrificed when tumors reached >15 mm in any direction.

For the orthotopic BC xenograft model, the abdomen was cleaned with an iodine solution, and a 1-cm midline incision was created to expose the bladder. A 28-gauge needle and a 1-ml disposable syringe were used for the injection of the cell suspension. The cells planned for inoculation were prepared at 2.5x10^4^ cells in 50 µl PBS and then mixed with 50 µl of Matrigel (Corning Matrigel Matrix High Concentration, Corning, NY) in a total of 100 µl cell suspension. The needle was inserted into the anterior muscular wall of the bladder, and 100 µl of cell suspension was carefully inoculated so as not to have any leakage to the surrounding region. The abdominal wound was closed in 2 layers with a 6/0 absorbable surgical suture. For in vivo bio-imaging experiment, 15mg/ml of D-Luciferin potassium salt (Sigma-Aldrich, USA) was dissolved in sterile distilled water. Mice were anesthetized using isoflurane, and 200 µl of D-luciferin solution was peritoneally injected, followed by the quantitative bioluminescence measurement using IVIS Lumina XRMS In Vivo Imaging System (Perkin Elmer, Waltham, MA).

*Clinical Data Sets and Statistical Analyses*

Clinical data sets were analyzed using Oncomine (https://www.oncomine.org/resource/login.html) and the cBio Cancer Genomics Portal (cBioPortal; www.cbioportal.org). Integrated Genome Viewer (https://www.broadinstitute.org/igv/) was used for visualization of ChIP-seq data analysis. Statistical analyses were performed using the unpair two-tailed Student’s t-test, one-way ANOVA, or two-way ANOVA with a post hoc Tukey’s honest significant difference (HSD) test using GraphPad PRISM (San Diego, CA). A p-value of less than 0.05 was regarded as statistically significant.
