# Supplementary figures and images for: Increased BUB1B/BUBR1 expression contributes to aberrant DNA repair activity leading to resistance to DNA-damaging agents
Source: Oncogene. 2021 Sep 20;40(43):6210–22. doi: 10.1038/s41388-021-02021-y (PMC8553621; doi:10.1038/s41388-021-02021-y)

Supplementary Figure 1

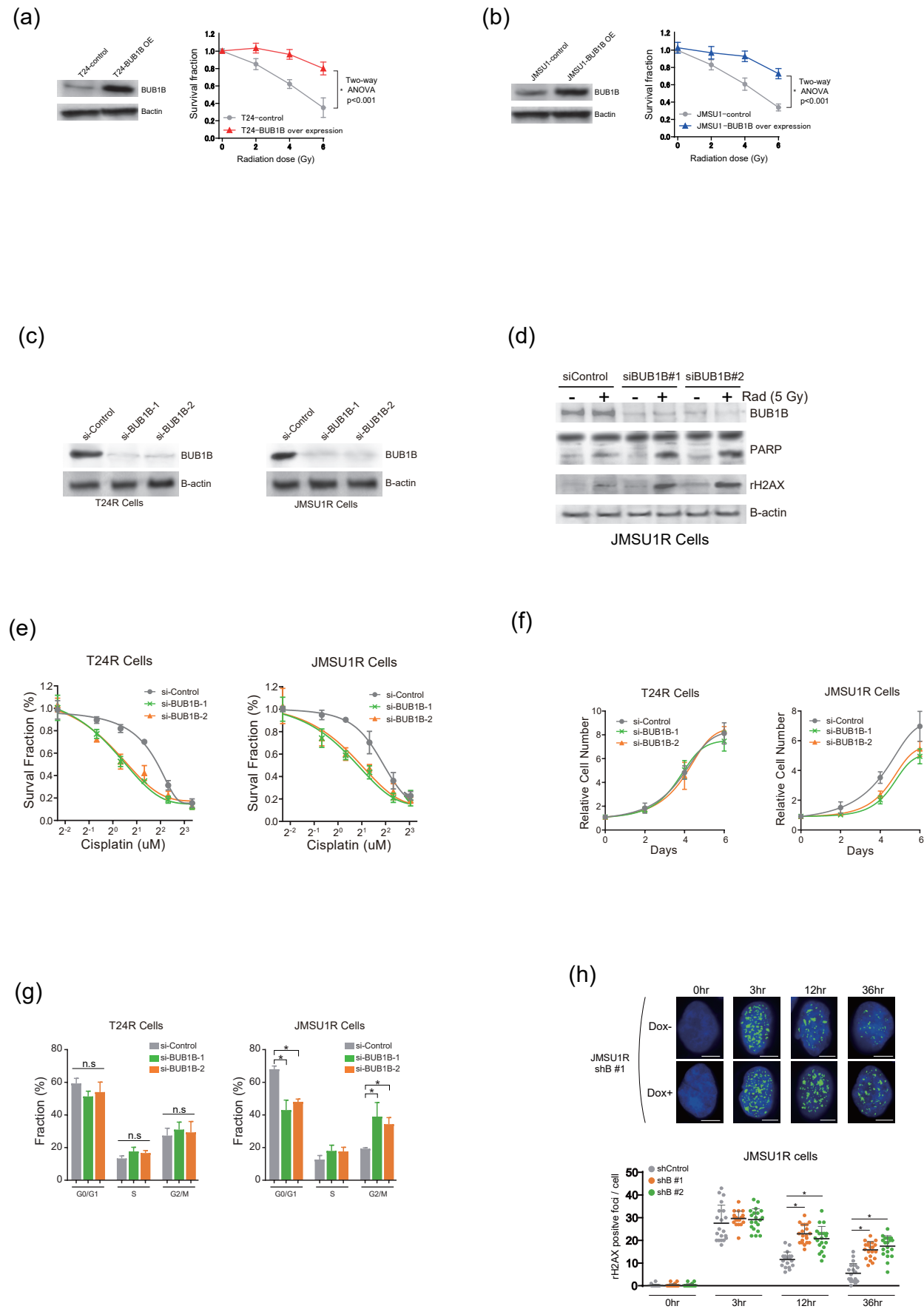

Supplement: Supplementary file 2 — Supplementary Figure 1 [file 41388_2021_2021_MOESM2_ESM.pdf]

Supplementary Figure 2

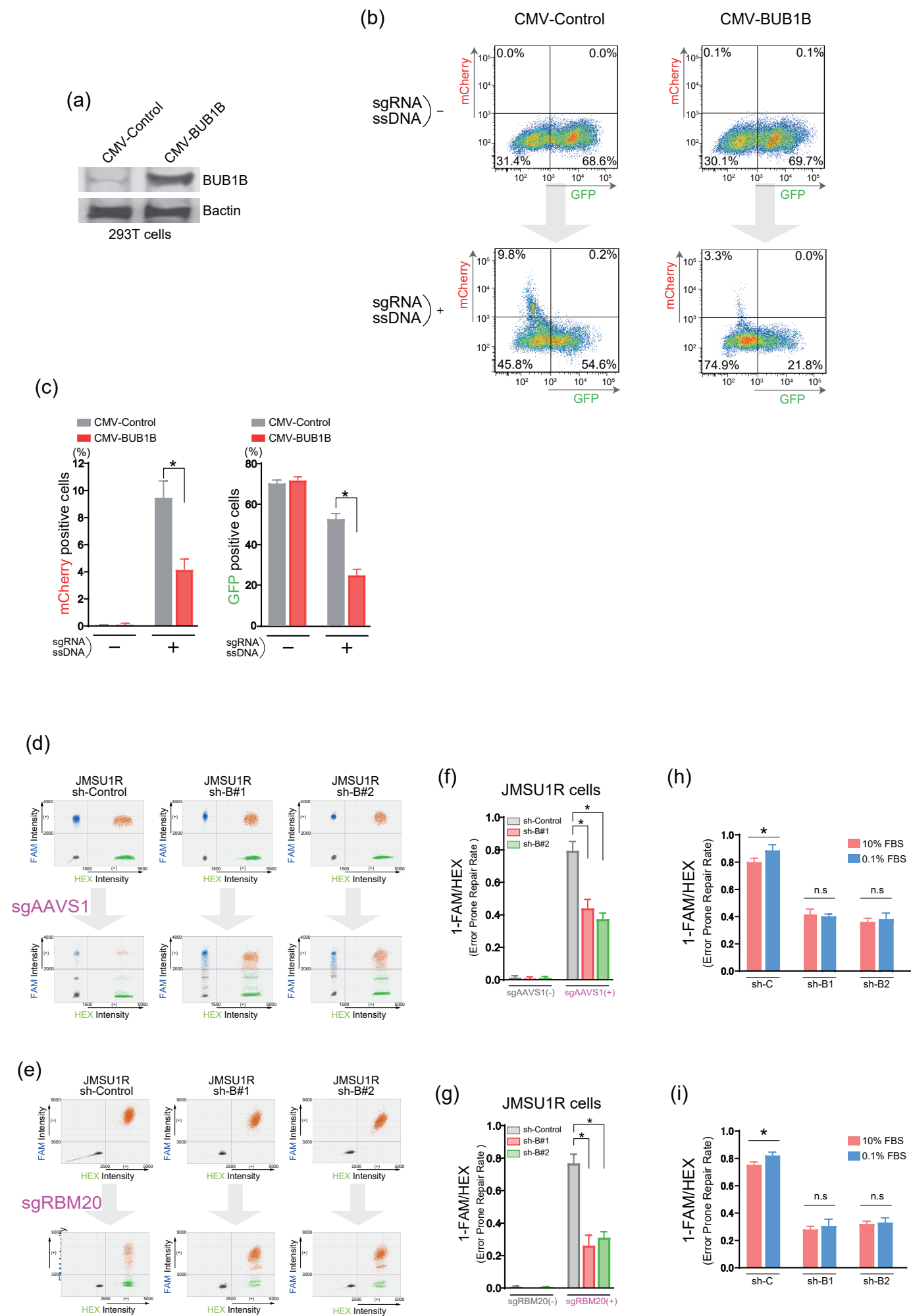

Supplement: Supplementary file 3 — Supplementary Figure 2 [file 41388_2021_2021_MOESM3_ESM.pdf]

Supplementary Figure 3

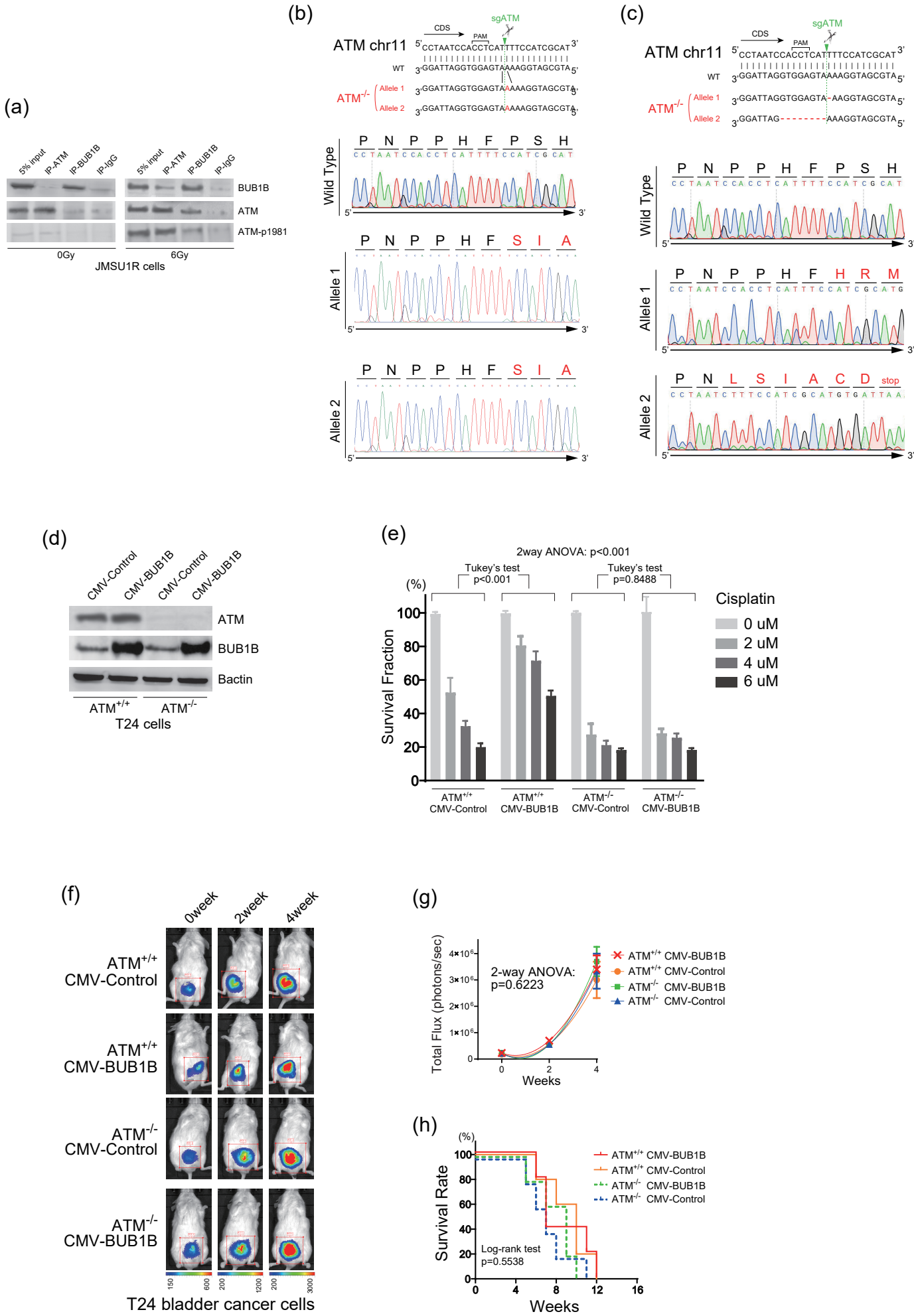

Supplement: Supplementary file 4 — Supplementary Figure 3 [file 41388_2021_2021_MOESM4_ESM.pdf]

Supplementary Figure 4

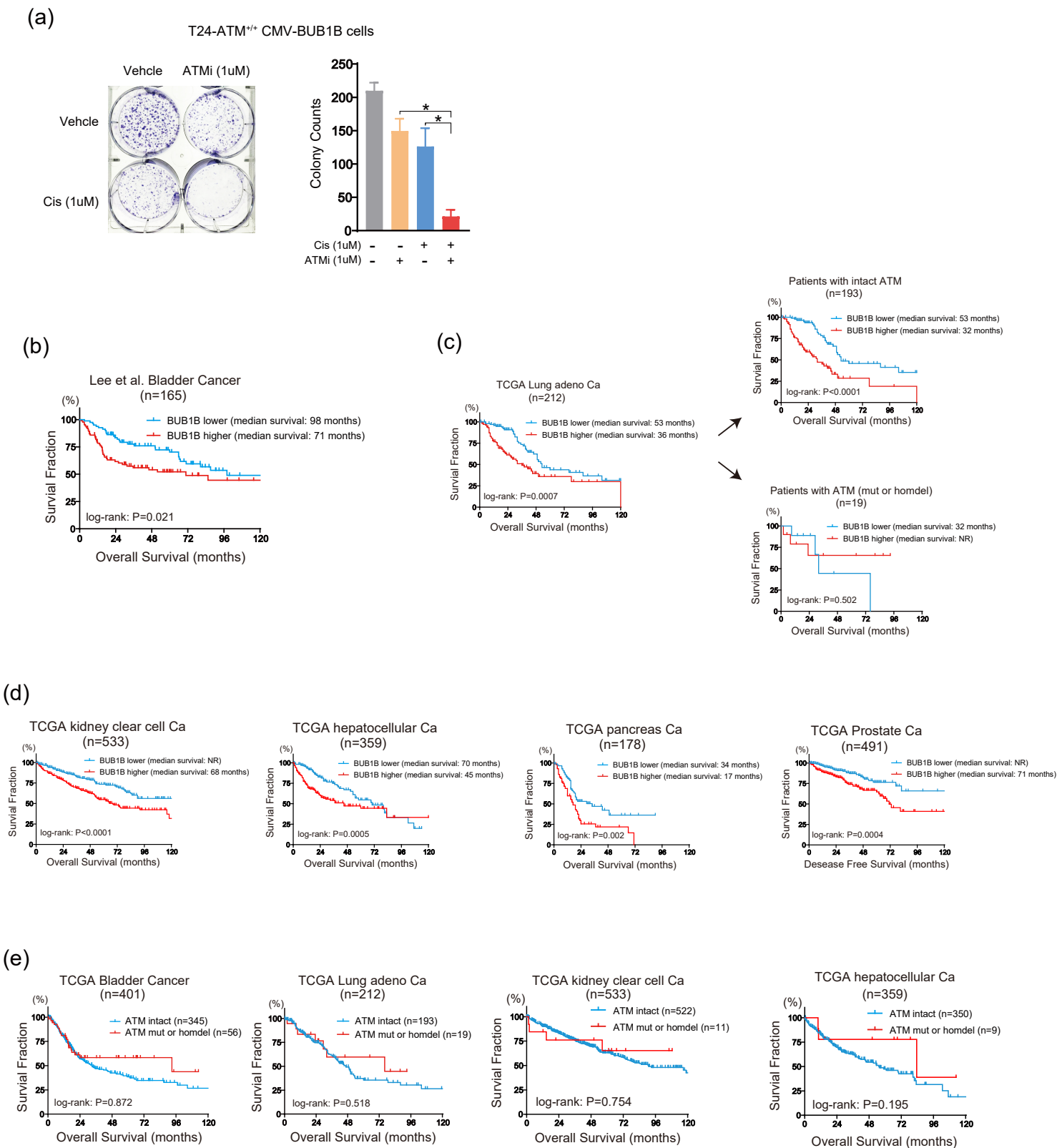

Supplement: Supplementary file 5 — Supplementary Figure 4 [file 41388_2021_2021_MOESM5_ESM.pdf]

## Supplementary Figure 5

(a)

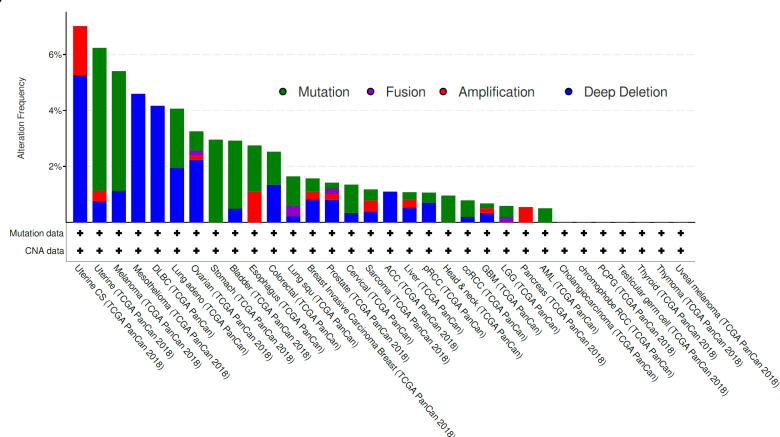

(b)

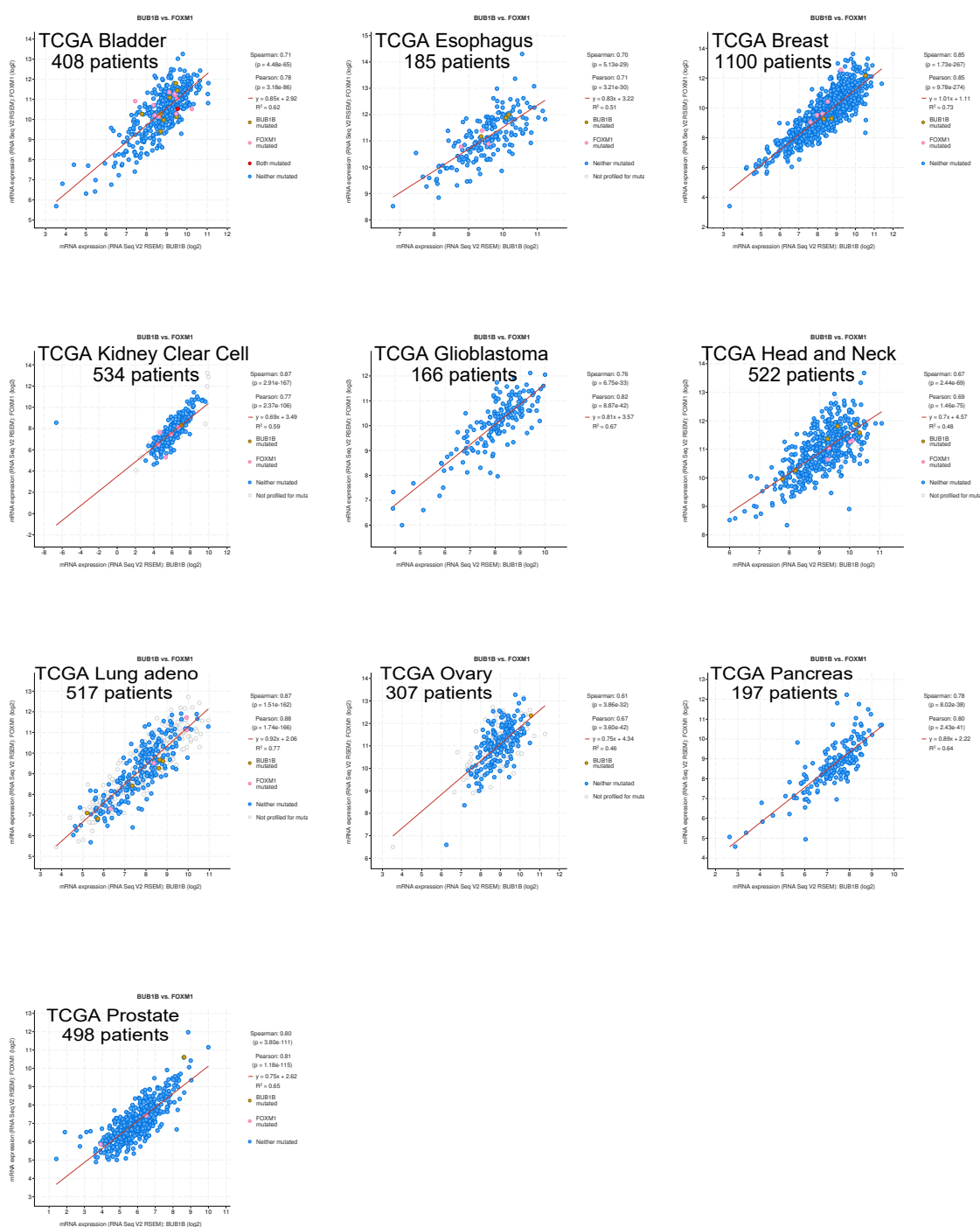

Supplement: Supplementary file 6 — Supplementary Figure 5 [file 41388_2021_2021_MOESM6_ESM.pdf]

Supplementary Figure 6

(a)

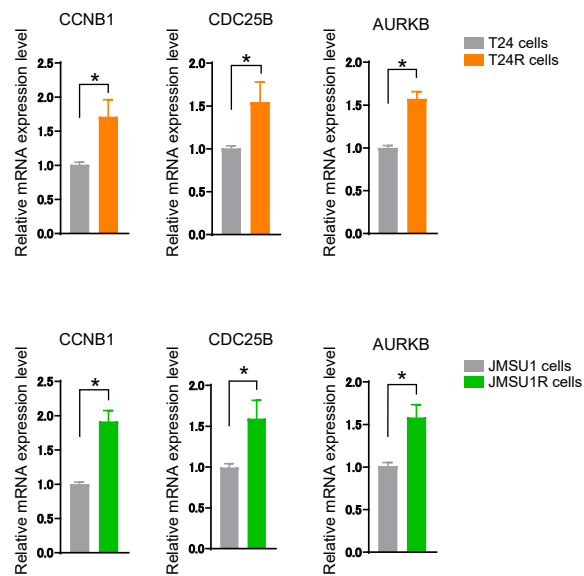

(b)

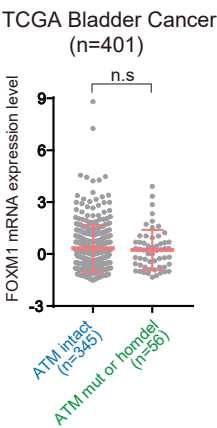

(c)

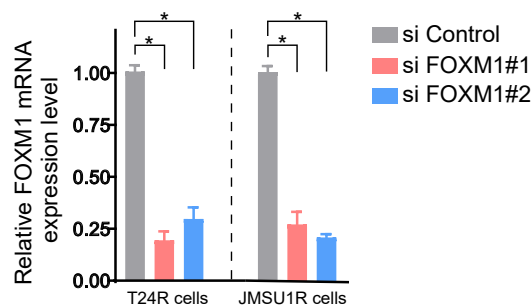

Supplement: Supplementary file 7 — Supplementary Figure 6 [file 41388_2021_2021_MOESM7_ESM.pdf]
