# Supplementary material for: Increased BUB1B/BUBR1 expression contributes to aberrant DNA repair activity leading to resistance to DNA-damaging agents
Source: Oncogene. 2021 Sep 20;40(43):6210–22. doi: 10.1038/s41388-021-02021-y (PMC8553621; doi:10.1038/s41388-021-02021-y)
Supplement: Supplementary file 8 — Legends of Supplementary Figures [file 41388_2021_2021_MOESM8_ESM.docx]

**Legends in Supplementary Figures**

Supplementary Figure 1: (a, b) Immunoblotting in indicated cell lines. B-actin was loaded as an internal control. BC cell lines were treated with IR in the indicated dose, followed by the measurement of cell viability assay after six days. The inhibitory effect on cell growth by the IR is presented as a relative value (mean ± SD) compared with control (0 Gy) as 100%. (c) Immunoblotting in indicated cell lines transfected with siRNAs. B-actin was loaded as an internal control. (d) Immunoblotting in indicated cell lines transfected with siRNAs three days before the IR treatment. Cells were collected 24 hours after the IR treatment. B-actin was loaded as an internal control. (e) The inhibitory effect on cell growth by cisplatin treatment with indicated concentrations was measured after 96 hours of treatment. Values are presented as a relative value (mean ± SD) compared with control as 100%. (f) Cell growth of indicated cells transfected with siControl and siBUB1B#1, and #2. (g) Cell cycle analysis in T24R and JMSU1R cells transfected with siControl and siBUB1B#1, and #2. Cells were collected for the analysis three days after the transfection. (h) Representative images of γ-H2AX-positive foci induced by 5 Gy of IR in JMSU1R sh-BUB1B#1 cells with or without 0.15 μg/ml of doxycycline. Scale bar indicates 10 μm. The bottom panels show the quantification of the number of γ-H2AX-positive foci among indicated cells cultured with 0.15 µg/ml of doxycycline. Results are shown as mean ± SD. * indicates p<0.05.

Supplementary Figure 2: (a) Immunoblotting in 293T cells with or without BUB1B stable overexpression. B-actin was loaded as an internal control. (b) Flow cytometry of GFP and mCherry positive 293T cells with or without BUB1B/BUBR1 stable overexpression. Cells were transfected with or without sgRNA targeting EGFP and single-stranded DNA template, followed by the analysis using flow cytometry three days after the transfection. (c) The proportion of GFP and mCherry positive cells in 293T cells with or without stable BUB1B overexpression. Results are shown as mean + SD. *P < 0.05, unpair t-test. (d, e) The result of ddPCR reporter assay in JMSU1R shControl and shBUB1B#1, 2 cells. Cells were cultured with 0.15 µg/ml of doxycycline for 72 hours, followed by the transfection of the CRISPR ribonucleoprotein (RNP) complex with or without sgRNA. Three days after the transfection, cells were harvested and analyzed. (f, g) The rate of consequences with mutagenic repair after the sgRNA transfection was defined as (1-FAM/HEX). Results are shown as mean + SD. *P < 0.05, unpair t-test. (h, i) The rate of mutagenic repair after the sgRNA transfection (defined as 1-FAM/HEX) in JMSU1R-sh C (control), B1 (BUB1B#1), and B2 (BUB1B#2). For the serum starvation, cells were cultured with 0.15 µg/ml of doxycycline (10% FBS) for 24 hours, then the medium was changed to 0.1% FBS including 0.15 µg/ml of doxycycline for 48 hours. Thereafter the transfection of CRISPR ribonucleoprotein (RNP) complex with or without sgRNA was performed in a 10% FBS medium. Results are shown as mean + SD. *P < 0.05, unpair t-test.

Supplementary Figure 3: (a) JMSU1R cells were treated with or without IR and incubated for 18 hours. Nuclear fractions were then collected and immunoprecipitated with antibodies specific to IgG, ATM, and BUB1B/BUBR1, followed by immunoblotting with indicated antibodies. (b) Sanger sequencing in T24R ATM^-/-^ cells generated by single-cell clonal isolation. (c) Sanger sequencing in T24 ATM^-/-^ cells generated by single-cell clonal isolation. (d) Immunoblotting of T24 ATM^+/+^ and ATM^-/-^ cells with or without stable BU1B/BUBR1 expression in indicated antibodies. B-actin was loaded as an internal control. (e) Cells were treated with cisplatin at the indicated concentration for 96 hours and then collected to measure the cell viability. Survival fraction was determined using the value without the treatment (0µM of cisplatin) in each cell as a control. Results are shown as mean ± SD. (f) Representative images of the quantitative luminescence measurement for each four groups in the orthotopic xenograft model without IR treatment. (g) Quantitative evaluation of the developed tumor in orthotopic xenograft mice. Total flux (photons/sec) in the region of interest (ROI) was recorded every two weeks. (h) Kaplan-Meier curves in the orthotopic mouse model of the indicated cells. A Log-rank test was performed to assess the survival difference.

Supplementary Figure 4: (a) Clonogenic survival assays in which T24-ATM^+/+^ CMV-BUB1B overexpression cells were plated to 6 well plates and treated with or without 1µM of ATM inhibitor (AZD0156) and cisplatin. The medium was changed every three days, and representative images are photographed after 21 days. The right panel shows the number of colonies counted in five random fields in 21 days. Results are shown as mean ± SD. * indicates p<0.05. (b) Kaplan-Meier curves in Lee’s BC data set [27] according to the BUB1B mRNA expression level. Patients were divided by the median cut-off of BUB1B mRNA expression level. A log-rank test was carried out to examine the survival difference. (c) Kaplan-Meier curves in TCGA lung adenocarcinoma data set [28] according to the BUB1B mRNA expression level. Patients were then stratified according to the ATM mutation status. In all the analyses, patients were divided by the median cut-off of BUB1B mRNA expression level. A log-rank test was carried out to examine the survival difference. (d) Kaplan-Meier curves, according to the BUB1B mRNA expression level in the kidney (clear cell carcinoma), hepatocellular carcinoma, pancreas carcinoma, prostate cancer. In all the analyses, patients were divided by the median cut-off of BUB1B mRNA expression level. A log-rank test was carried out to examine the survival difference. (e) Kaplan-Meier curves according to the ATM mutation status in BC, lung adenocarcinoma, kidney cancer (clear cell carcinoma), hepatocellular carcinoma. A log-rank test was carried out to examine the survival difference.

Supplementary Figure 5: (a) The status of mutation and copy number alteration in BUB1B/BUBR1 among various types of cancer. Data is visualized by cBioPortal. (b) The correlation between BUB1B/BUBR1 and FOXM1 mRNA expression level in various types of cancers. Data are visualized using cBioPortal.

Supplementary Figure 6: (a) The quantitative PCR for the mRNA expression level of CCNB1, CDC25B, AURKB in indicated BC cells. Data are shown as mean ± SD. * indicates p<0.05. (b) FOXM1 mRNA expression level according to the ATM mutation status in TCGA BC dataset (n=401) [10]. (c) Result of quantitative PCR in T24R and JMSU1R BC cells. Cells were transfected with indicated siRNAs and then collected for the analysis 48 hours after the transfection. Data are shown as mean ± SD. * indicates p<0.05.
